# Managing the Unmanageable: Understanding the Competing Effects of Salinity and Nutrient Loading on Estuarine Water Quality

**DOI:** 10.1007/s12237-026-01792-5

**Published:** 2026-08-04

**Authors:** Marcus W. Beck, Edward T. Sherwood, Rebecca R. Murphy, Kerry Flaherty-Walia, Qian Zhang, Blake A. Simmons, Maya C. Burke

**Affiliations:** 1 Tampa Bay Estuary Program, 263 13th Ave S., Suite 350, St. Petersburg, FL 33701 USA; 2 University of Maryland Center for Environmental Science/U.S. Environmental Protection Agency Chesapeake Bay Program, 1750 Forest Drive, Annapolis, MD 21401 USA

**Keywords:** Chlorophyll-a, Generalized Additive Models, Nutrients, Salinity, Tampa Bay

## Abstract

Changing climatic conditions that can alter physical characteristics of surface waters may limit the effectiveness of conventional approaches to nutrient management. This study evaluated the competing effects of nutrient loads and changing salinity on chlorophyll-a dynamics in Tampa Bay (Florida, USA). This estuary has demonstrated long-term recovery of water quality primarily through wastewater infrastructure improvements, whereas a gradual freshening of the bay over the last few decades may compromise future management efforts to protect water quality. The relative effects of loading and salinity over a forty-year time period were evaluated using normalized predictions from Generalized Additive Models (GAMs). All bay segments showed a gradually increasing influence of reduced salinity on chlorophyll-a. Old Tampa Bay in the northwest also showed an increasing influence of nutrient loading. Simulation scenarios using the GAMs assessed the relative likelihood of exceeding regulatory chlorophyll-a standards under future salinity conditions. All bay segments showed an increased likelihood of exceeding regulatory standards, particularly in Old Tampa Bay where likelihood increased by ~ 16% over a fifty-year simulation. The results suggested that current nutrient loads should be substantially reduced below the federally-recognized Total Maximum Daily Load to maintain likelihood of meeting regulatory chlorophyll-a standards in the future that is similar to present. By understanding the effects of largely unmanageable characteristics of the bay, water quality managers can reassess efforts on limiting factors they can control relative to those they cannot.

**Supplementary Information:**

The online version contains supplementary material available at https://doi.org/10.1007/s12237-026-01792-5.

## Introduction

Water quality management has been guided for decades in the United States by frameworks established under the 1972 amendments to the Federal Water Pollution Control Act (Clean Water Act, e.g., Keller & Cavallaro, 2008). Similar mandates under the European Water Framework Directive define processes for identifying and remediating unacceptable water quality conditions (e.g., Hering et al., 2010). While these legislative frameworks have provided the foundation for thousands of efforts to protect and improve water quality, few examples exist that demonstrate the effectiveness of these approaches at large scales given the challenges of collaborative management across broad regions (Adler & Landman, 1993; Moss & Newig, 2010). Successful management paradigms require intentional efforts that bridge multiple entities and also consider the unique setting of each waterbody to create a nuanced and iterative approach to adaptively manage water quality (Cloern, 2001; Testa et al., 2025). In locations where past efforts have been successful to remediate environmental degradation, understanding what has been effective in the past, and if it will continue to be effective in the future, is critical for long-term protection of the resource (Paerl et al., 2006).

Case studies to assess questions on long-term management effectiveness are only possible when sufficient data exist and ecosystem response to management actions has been demonstrated (Greening et al., 2014; Schiff et al., 2016). Tampa Bay (Florida, USA) provides an exceptional opportunity to evaluate these questions. First, Tampa Bay has one of the longest surface water quality monitoring programs in the United States. These data have been used for many research applications (Morrison et al., 2006; Beck & Hagy, 2015; Karlen et al., 2023) and have also contributed to the development of regulatory standards for different parts of the bay (Janicki & Wade, 1996; Janicki et al., 2000). Similarly, monthly long-term estimates of external nutrient inputs are also available and have been used to monitor the effectiveness of management interventions to reduce excess loads to the bay (Greening & Janicki, 2006; Janicki et al., 2023). Second, Tampa Bay represents a known success story where large-scale ecosystem recovery has been documented from concerted efforts to reduce external nutrient loads, which was highly dependent on a robust management paradigm and the cooperative efforts of multiple entities (Sherwood et al., 2016). Recent changes in the bay’s ecology have brought into question the continuing effectiveness of this paradigm, in part due to changing load sources over time as the human presence in the watershed continues to grow. Additionally, long-term effects of climate change have also altered the bay’s ecology, with documented changes in water temperature and salinity (Beck et al., 2024). Large-scale effects of physical drivers can alter the bay’s assimilative capacity for external nutrients, bringing into question the effectiveness of existing management paradigms and protective thresholds that were developed decades ago when the bay’s ecology was much different. This study provides a quantitative assessment of the relative risk of exceeding regulatory standards under future climate change.

This work evaluates the competing associations of nutrient loading and salinity with Tampa Bay’s water quality over four decades. The association between water quality and external nutrient inputs is well-understood and comparatively straightforward (i.e., Nixon, 1995). Nitrogen, in particular, is a well-documented limiting nutrient for phytoplankton growth in Tampa Bay because of the underlying geology of central Florida that influences the chemical characteristics of surface waters and its dissolved content via groundwater flow (Wang et al., 1999; Yates et al., 2011). Salinity gradients and mixing dynamics in estuaries have also received substantial attention in coastal research given the fundamental influence on biogeochemical processes in estuaries (Bowden, 1963; Kuo & Neilson, 1987; Damme et al., 2005). However, the role of salinity in eutrophication is not as straightforward as nutrients, since it reflects characteristics of the physical environment that do not directly control primary production in the same manner as macro-nutrients (McCorquodale et al., 2009; Lui & Chen, 2012). Salinity is used herein as a proxy for physical factors that may affect phytoplankton community dynamics, such as through tidal mixing, residence time, freshwater inputs, or other factors related to circulation. Thus, we are not concerned with the direct effect of salinity on algal dynamics, rather we seek an understanding of how changing physical conditions could change the ecology of the bay through the lens of changing salinity. Such changes potentially alter the bay’s response to external nutrients, which can be managed through substantial efforts, whereas salinity at large estuary scales cannot be as effectively managed by similar means (Paerl et al., 2006).

Models using mechanistic and spatially-explicit representations of estuarine processes have been leveraged extensively for regulatory assessments and to evaluate the potential effectiveness of management interventions (Borsuk et al., 2002; Borah et al., 2006; Sherwood et al., 2015). While these tools will continue to serve this valuable role, empirical models can provide rapid and more lightweight approaches to test similar scenarios or to develop an alternative understanding of system dynamics. In fluvial environments, the Weighted Regressions on Time, Discharge, and Season (WRTDS) method has been used extensively to model long-term trends in nutrient concentration and flux (Hirsch et al., 2010). Central to this approach is the inclusion of the confounding effects of discharge on trend analysis. In estuarine settings, salinity can be used as a conservative tracer in WRTDS to account for the effects of freshwater inputs or circulation (Beck & Hagy, 2015). These approaches allow an estimate of flow- or salinity-normalized trends in water quality parameters by removing their effects on the predictions. Generalized Additive Models (GAMs) have also been used in this capacity, while allowing flexibility to include additional parameters and more accessible measures of uncertainty (Wood, 2017; Murphy et al., 2019; Schramm, 2023). We adopt GAMs for this analysis based on these advantages, while also recognizing that the WRTDS approach has been foundational in these approaches to normalization. In particular, we use model predictions and differences with the normalized results to evaluate the relative effects of different drivers on chlorophyll-a dynamics.

Reporting and management of external nutrient loads to Tampa Bay have been the responsibility of the Tampa Bay Nitrogen Management Consortium (TBNMC) since the early 2000s. These efforts are guided by a state Reasonable Assurance Plan whereby compliance assessments and water quality improvement efforts are reported to the Florida Department of Environmental Protection to address a federally-recognized Total Maximum Daily Load (TMDL) for nitrogen. Regulatory criteria for chlorophyll-a apply to each bay segment, such that exceedances for more than two consecutive years require review of a bay segment’s assimilative capacity. The assimilative capacity for each bay segment is currently defined by a maximum annual nitrogen loading beyond which water quality outcomes are likely detrimental to seagrasses. Similarly, chlorophyll-a thresholds for each bay segment were based on nitrogen loading to maintain a light environment supportive of seagrass growth (Janicki & Wade, 1996). TBNMC investments to manage external nutrient loads have contributed to the long-term recovery of Tampa Bay. However, current physical conditions and the bay’s ecological response compared to past conditions from which regulatory thresholds were developed have been the focus of recent discussions by the TBNMC and serve as a primary motivator for this current work. Chlorophyll-a and total nitrogen load thresholds for each bay segment are evaluated using GAMs for scenario assessments under expected future changes and management actions.

The objectives of this study were to (1) develop Generalized Additive Models (GAMs) for chlorophyll-a trends for the four major bay segments of Tampa Bay as a function of time, salinity, and total nitrogen loading, (2) quantify the relative influence of salinity changes and nitrogen loading on chlorophyll-a trends, and (3) assess the potential likelihoods of exceeding regulatory criteria under future salinity changes and different loading scenarios. We hypothesize that water quality drivers have changed over time, warranting a shift in thinking about management strategies to account for the effects of factors that are difficult to control (salinity) under conventional management paradigms. This shift may be particularly important in upper bay segments where long-term nitrogen loads have changed, residence time is affected by freshwater inflows and circulation, and current loading thresholds may be insufficient to protect water quality under future expected changes in salinity. We focus primarily on salinity and not additional climate change stressors (i.e., temperature) given that Old Tampa Bay (northwest segment) has circulation dynamics that contributed to regulatory exceedances (Sherwood et al., 2015; Luther & Meyers, 2022) and salinity can serve as a proxy to better understand these effects. Future analysis of temperature effects could be beneficial. Overall, this work provides an in-depth assessment of chlorophyll-a trends for informing the management of water quality under past and contemporary conditions, with a generalized framework that could be applied in other locations with long-term data.

## Methods

**Fig. 1:**
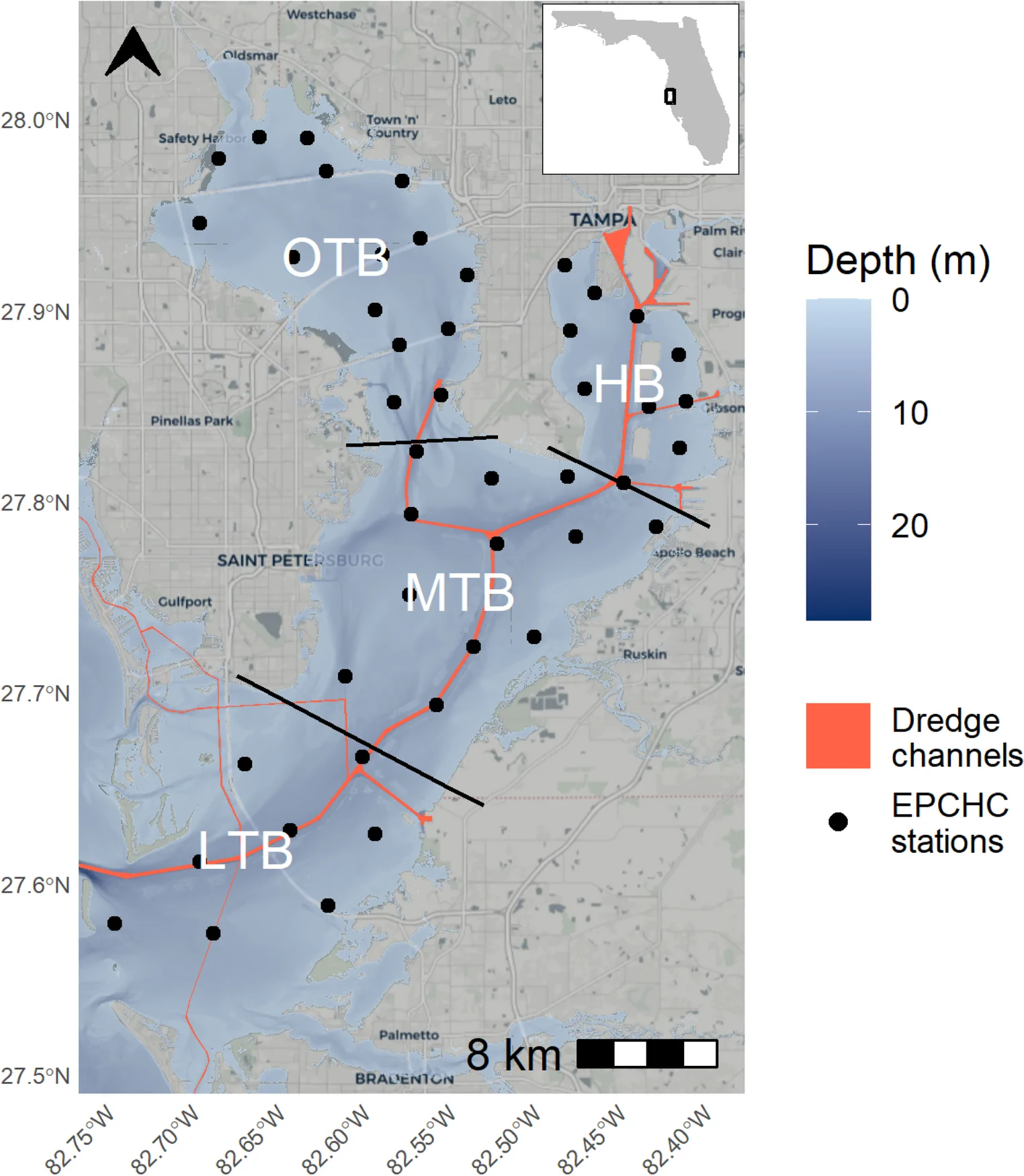
Tampa Bay on the west coast of Florida, USA. Bay segments are denoted by their labels and boundaries. Red lines show major dredged areas, including the main shipping canal from the Gulf to Hillsborough Bay. Points show long-term monitoring stations from the Environmental Protection Commission of Hillsborough County (EPCHC) where chlorophyll-a and salinity data were used for modeling. OTB: Old Tampa Bay; HB: Hillsborough Bay; MTB: Middle Tampa Bay; LTB: Lower Tampa Bay

Tampa Bay on the west coast of Florida is a sub-tropical shallow estuary covering 400 mi^2^ (1036 km^2^) of open water that includes native habitats of seagrasses, macroalgae, oysters, tidal flats, and hard bottom (Fig. 1). Average depth of the bay is 11 ft (3.4 m) with a maximum depth of 43 ft (13.1 m) in the dredged shipping channels that primarily traverse the longitudinal axis. The water column is partially to well-mixed and typically does not exhibit strong stratification (Chen et al., 2019). The bay is divided into four major segments defined by physical and natural boundaries; Old Tampa Bay (OTB) in the northwest, Hillsborough Bay (HB) in the northeast, Middle Tampa Bay (MTB), and Lower Tampa Bay (LTB) at the southernmost extent that exchanges with the Gulf of Mexico (Fig. 1). The watershed is 2200 mi^2^(5872 km^2^) and includes an extensive human presence, with 42% of the land as urban or suburban (Southwest Florida Water Management District, 2025). Agricultural and mining activities cover about 25% of the watershed, whereas the remainder includes native habitats of hardwood or scrub uplands and freshwater wetlands.

Residence time varies with inflow, wind, and spatial scale. Baywide estimates range from 75 (Eulerian) to 159 (Lagrangian) days (Burwell et al., 2000; Morrison & Yates, 2011), with substantial variation by location. Major inflows enter HB via the Hillsborough and Alafia Rivers, MTB via the Little Manatee River, and LTB via the Manatee and Braden Rivers. LTB, with direct Gulf exchange, has the shortest residence time of a few days, whereas OTB and HB have the longest. However, HB flushes relatively rapidly compared to OTB due to major freshwater inflows and enhanced baroclinic circulation via the main dredge channel (Fig. 1) that extends from the mouth of the bay to upper HB (Morrison & Yates, 2011). More importantly, OTB has three major bridges with substantial causeways that are known impediments to circulation (Luther & Meyers, 2022). Consequently, residence times in OTB can last as long as three months (Janicki Environmental, Inc., 2015).

Long-term external nutrient loading from multiple sources has decreased overall in Tampa Bay over the last fifty years (Janicki et al., 2023). Point sources have been substantially reduced following upgrades of wastewater plants to advanced tertiary treatment, resulting in an overall nitrogen load reduction of approximately two thirds from the 1970s to present for the entire bay (Zarbock et al., 1994; Janicki et al., 2023). Conversely, nonpoint sources primarily from stormwater runoff remain the dominant source of nutrients to the bay, particularly in OTB and HB. Loading from nonpoint sources varies substantially between years with changes in rainfall and with more long-term climatic drivers (i.e., El Niño vs. La Niña years). However, a weak but long-term increase in rainfall over the past fifty years has led to gradual increases in stormwater loads and, consequently, a freshening of Tampa Bay, particularly in the upper bay segments (Beck et al., 2024). Overall, HB receives the largest external nitrogen loads on an annual basis, followed by MTB, OTB, and LTB, respectively (Beck, 2022).

### Data Processing

**Fig. 2:**
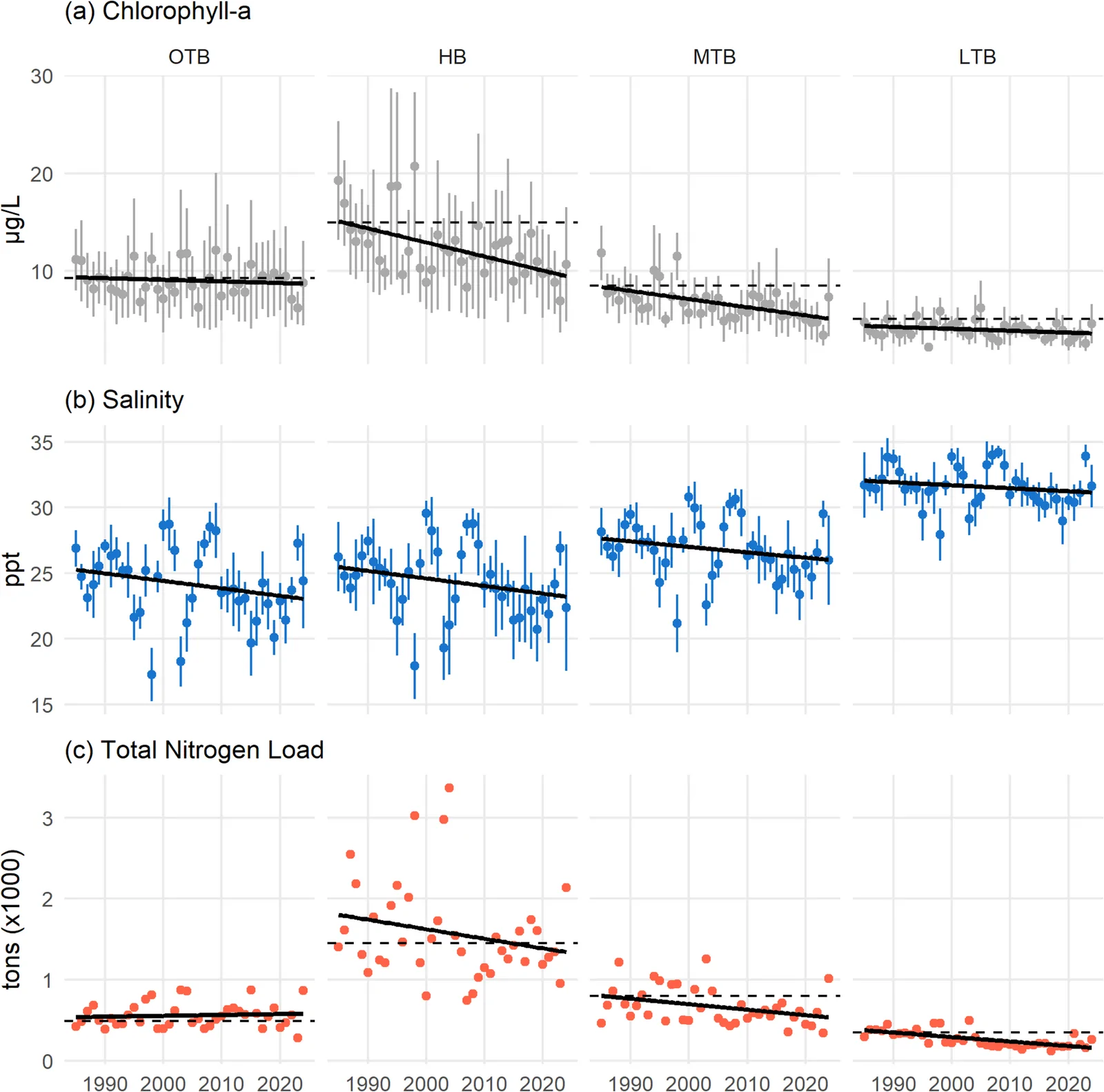
Annual chlorophyll-a (**a**), salinity (**b**), and total nitrogen load (**c**) for each bay segment from 1985 to present. Chlorophyll-a and salinity are shown as annual averages with 95% confidence intervals and the total nitrogen loads are the annual sums across each month. Relevant regulatory standards are shown as dashed lines for chlorophyll-a and total nitrogen load. Linear trends are also shown with the solid black lines. OTB: Old Tampa Bay; HB: Hillsborough Bay; MTB: Middle Tampa Bay; LTB: Lower Tampa Bay

Long-term monitoring data for Tampa Bay were obtained from the Environmental Protection Commission of Hillsborough County (EPCHC). This monitoring program has been ongoing since the mid-1970s, with monthly sampling at 45 fixed stations across the major bay segments (Fig. 1). Surface chlorophyll-a and salinity observations at each station were averaged by month for each bay segment (annual averages by bay segment are shown in Fig. 2a, b). Salinity is measured at surface, mid, and bottom depths at each station, whereas only surface salinity was used for analysis given similar sampling depth as chlorophyll-a. Further, initial analyses using depth-averaged salinity produced models with nearly identical performance when compared to those using surface salinity. These similarities were not unexpected given that the majority of Tampa Bay is shallow, well-mixed, and typically does not stratify, as noted in the prior section. Salinity and chlorophyll-a gaps were filled using linear interpolation between observed values, where the maximum gap filled was three months. Salinity and chlorophyll-a gaps accounted for less than 5% and 2%, respectively, of the observations for each bay segment. Monthly data from 1985 to present by bay segment were used for analysis to coincide with the period of record for the nutrient loading data (* n* = 480 for each bay segment).

Monthly total nitrogen loading for each bay segment from 1985 to present was estimated following methods in Zarbock et al. (1994; additional details in Janicki Environmental, Inc., 2023). In brief, loading data were estimated as tons per month by external source, including domestic point-sources (wastewater treatment effluent, reclaimed water), industrial point sources, non-point sources, atmospheric deposition, groundwater/springs, and material losses from nutrients entering the bay through port/shipping activities (annual totals by bay segment are shown in Fig. 2c). Point source loads were estimated from reported flow and concentration data for every large permitted entity (output > 0.1 million gallons per day) in the Tampa Bay watershed. Conversely, non-point source loads were estimated from gauged and ungauged portions of the watershed by combining rainfall and flow estimates with observed water quality data or estimated from event mean concentration data by land use and soil type. Atmospheric deposition was estimated using rainfall and airshed chemical constituent data obtained from the Verna well-field long-term air quality monitoring site (NTN Site FL41, NADP, 2022). Groundwater loading was estimated from aquifer concentration data combined with potentiometric sub-surface flow data, whereas springs loadings were calculated directly from discharge and concentration data measured at each spring (downstream of gauged non-point source loading sites). Finally, material losses were based on a loss factor per unit fertilizer shipped at each port location. For the purpose of this analysis, no distinction was made between load sources, such that the total load as the sum across sources was used for modelling. However, we note that trends by sources have varied by location, e.g., non-point source loads have increased in OTB, whereas point sources have decreased (Janicki et al., 2023).

### GAMs

Four separate GAMs were created for each bay segment using the combined water quality and loading data for the monthly observations from 1985 to present. The primary goal was to develop models with sufficient explained variance for chlorophyll-a by including the effects of salinity, nitrogen loading, and time. Time was expressed as two separate variables for each time step, first as continuous decimal time (e.g., July 1st 2017 is 2017.5) and as day of year to capture a seasonal component (Murphy et al., 2019). Loading was evaluated during the same month of the chlorophyll-a sample and as cumulative monthly sums up to three months prior to the month of observation. Previous work has suggested cumulative monthly sums of preceding loading may better explain variation in chlorophyll-a (Janicki & Wade, 1996). Each predictor was evaluated as an additive smooth to quantify an individual association with chlorophyll-a, whereas interaction smoothers were also included for every pairwise combination of the predictors. Model structure was evaluated using the “select” option from the mgcv R package (Wood, 2017), which allows the influence of individual smoothers to be removed by penalizing them to zero if they have negligible effects. Comparisons of the full models with those using the select option showed minimal differences (Table S1, using differences in Akaike information criterion or AIC, ∆AIC < 3 for all models, Akaike, 1974) and the full models are used throughout. Using the full models also ensures the same model structure for describing drivers and outcomes of scenario testing between bay segments, as desired for objectives (2) and (3). This also allowed direct comparability between models regarding explained variance, either as a whole or for individual predictors.

The model notation was:

$$\:\begin{array}{rc}chla_i&\:\sim\:Gamma\left({\mu\:}_i,\phi\:\right)\\\:\log\left({\mu\:}_i\right)&\:={\beta\:}_0+f_1\left(dec\_time_i\right)+f_2\left(doy_i\right)+f_3\left(sal_i\right)+f_4\left(load_i\right)+\\\:&\:\:+f_{12}\left(dec\_time_i,doy_i\right)\\\:&\:\:+f_{13}\left(dec\_time_i,sal_i\right)\\\:&\:\:+f_{14}\left(dec\_time_i,load_i\right)\\\:&\:\:+f_{23}\left(doy_i,sal_i\right)\\\:&\:\:+f_{24}\left(doy_i,load_i\right)\\\:&\:\:+f_{34}\left(sal_i,load_i\right)\end{array}$$

where chlorophyll-a was modelled as a Gamma-distributed response variable with mean $\:{\mu\:}_{i}$ and dispersion parameter ϕ for observation * i* (year-month). A Gamma distribution for the model errors was used to account for an increase in variance with the mean, which is a reasonable expectation for chlorophyll-a data. Models using a Gaussian distribution with a log-link function were also evaluated, although using a Gamma distribution was superior when compared with AIC (Table S2; Akaike, 1974). The log-transformed mean chlorophyll-a was modelled as an intercept ($\:{\beta\:}_{0}$) plus smooth functions of decimal time (dec_time), day of year (doy), salinity (sal), and loading (load), along with tensor product smooth interactions between each pair of the four predictors. Specifically, $\:{f}_{1}$() is a thin plate regression spline with k = 40 basis functions, $\:{f}_{2}$() is a cyclic cubic regression spline with k = 10 basis functions, $\:{f}_{3}$() and $\:{f}_{4}$() are thin plate regression splines with k = 10 basis functions, and $\:{f}_{12}$(), $\:{f}_{13}$(), $\:{f}_{14}$(), $\:{f}_{23}$(), $\:{f}_{24}$(), and $\:{f}_{34}$() are tensor product smooths (excluding main effects) with k = (5, 5) basis functions (25 total). Smoothing parameters were estimated using restricted maximum likelihood using the gam() function from mgcv (Wood, 2017), which can further impose constraints by penalizing “wiggliness” for smoothers that minimally influence the results (Wood, 2011).

Different forms of the predictors and smoothers were evaluated to determine the best model fit for each bay segment (following Murphy et al., 2021). First, the K values (the upper limit on the basis dimensions) for each smoother were chosen to allow sufficient degrees of freedom to describe the association of each predictor with chlorophyll-a. An alternative approach where the K values were restricted for the decimal time smoother was also evaluated to allow additional variation to be fit for the other predictors (i.e., constraining the amount of “wiggliness” that may be attributed to time alone so that salinity or loading can better describe chlorophyll-a). Second, the salinity and loading variables were seasonally corrected by taking the residuals of a separate GAM for each predictor compared to day of year. This correction was made to isolate a signal independent of seasonal effects. The correction for loading also included a fit to the corrected salinity time series to remove any variation attributed to the latter. Finally, loading lags in each model were evaluated by using loading at the month of observation (no lag) up to a cumulative lag of three months.

Once models were fit, the gam.check() function was used to compare the estimated degrees of freedom for each smoother to the maximum degrees of freedom allowed by the K value. None of the smoothers were within 80% of the maximum degrees of freedom allowed, suggesting sufficient flexibility to describe the association. ACF plots (Venables & Ripley, 2002) also showed minimal autocorrelation of model residuals at lag 1 (all < 0.20 $\:\rho\:$), indicating a time-correlated variance structure was not needed for the models. Multicollinearity (identifiability) among model predictors was evaluated with the concurvity() function.

Among the models evaluated, the simplest were chosen that did not include alterations to the predictors (seasonal correction or loading lags), nor constraints on the K values. Initial analyses indicated minimal improvements in model fit with these alterations (Table S3). For all models, multicollinearity was high for the individual smoothers (approaching 1), although this is common when interactions are included (Wood, 2017). Multicollinearity of the interaction smoothers was lower for the simpler models (Tables S4-S7), but still relatively high on a scale of 0 to 1. This does not preclude the use of these models for prediction, rather caution should be used to interpret the individual contribution of each smoother. The remainder of the text uses the simpler models.

In addition to predicted chlorophyll-a, the GAMs were used to create two additional types of predictions. First, salinity-normalized predictions were made by predicting chlorophyll-a across a range of salinity values (minimum and maximum observed over the period of record for each bay segment, 1985–2024) at each time step, then taking the mean of those predictions as the salinity-normalized value (Figure S1). This produces a time series that can be considered the chlorophyll-a trend independent of changes in salinity (i.e., just time and loading). The approach is consistent with other studies examining the effects of salinity on chlorophyll-a concentrations (Beck & Murphy, 2017; Murphy et al., 2019).

Salinity-normalized predictions at mean loading were also estimated. These predictions can be considered the chlorophyll-a trend independent of changes in both salinity and loading. This is accomplished by predicting chlorophyll-a at each time step using the mean loading (over the period of record for each bay segment) with the salinity-normalized predictions for each bay segment across the entire time series (Figure S1). Mean loading is used in this case as opposed to the more involved grid based approach for salinity normalization. This was preferred for ease of interpretation and absence of a typical seasonal cycle that would be expected for a variable like salinity.

Although the effects of salinity and loading can be inferred from conventional GAM summaries, either by evaluating tabular model output or from effects plots, they do not provide a holistic interpretation that can be obtained from the normalized results. By utilizing the model predictions that consider the effects of all modeled parameters and normalizing the output as described above, the unique effects of salinity and loading can be more clearly evaluated, especially over time. This approach is essentially a GAM-equivalent to the normalization from other methods (e.g., WRTDS, Hirsch et al., 2010; Beck & Hagy, 2015).

Finally, the models for each bay segment were evaluated for fit using conventional GAM summary statistics, which included Generalized Cross-Validation scores, explained deviance, estimated and reference degrees of freedom, F, and p-values for each smoother. Simple correlations between observed and predicted chlorophyll-a were also evaluated for different year groupings (1985–1998, 1999–2011, 2012–2024, as an approximate split of the period of record into equal groups) and by season (Wet: June to September; Dry: October to May) for each year grouping to assess when model fit may differ relative to the entire time series.

### Partitioning Drivers of Chlorophyll-a Change

Objective (2) was to assess the relative effects of salinity and loading on chlorophyll-a trends from the GAMs for each bay segment. This was accomplished by comparing the predicted values from the GAMs with those from the salinity-normalized predictions and the salinity-normalized predictions at mean loading. Specifically, the difference between the predictions and salinity-normalized values as a simple subtraction was used as an expression of salinity effects on chlorophyll. Likewise, the difference between the salinity-normalized values and the salinity-normalized values at mean loading was used as an expression of the loading effects on chlorophyll (Figure S1c, inset).

The differences among the predictions by bay segment were assessed as simple visualizations of the annual results over time and using a state-space diagram plotting the differences for each time series over time on separate axes. For the latter, the x-axis showed the salinity effects and the y-axis showed the loading effects, both in $\:\mu\:$g/L to show the relative contribution to chlorophyll-a per unit change in salinity or loading. The values on each axis can be interpreted relative to the long-term changes in both loading and salinity, where generally both loading and salinity have decreased over time. The direction (plus or minus) of chlorophyll-a change on each axis represents a response to these long-term changes in both drivers. Individual years were plotted on the diagram to assess the relative effects of salinity changes and/or loading on the relative chlorophyll-a changes each year. Depending on where a year is located on the plot, conclusions can be made on which factor (or both) may or may not have influenced chlorophyll-a each year. Additionally, these differences were further evaluated for different year groupings (1985–1998, 1999–2011, 2012–2024) and by season (Wet, Dry) within each year grouping to further evaluate periods of time when salinity and/or loading had differing effects on chlorophyll-a.

### Likelihood of Exceeding Regulatory Criteria

Objective (3) was to use the GAMs to assess the likelihood of exceeding regulatory criteria under projected (future) salinity changes and loading scenarios. This was accomplished using the simulate() function from the gratia R package (Simpson, 2025), which predicts randomized GAM outputs within the prediction interval of the model based on user inputs. For each scenario, 10,000 simulations were used to estimate chlorophyll for different salinity and loading scenarios, where the mean and standard deviation of the percent of years exceeding the thresholds were used to identify the likelihood of exceeding regulatory criteria over time. Herein, “over time” is defined as a hypothetical period from present to fifty years in the future at one year time steps based on potential salinity changes over this period and constant loading for different scenarios (current, at the TMDL, half the TMDL, and double the TMDL). Chlorophyll-a regulatory thresholds for each bay segment are 9.3, 15.0, 8.5, and 5.1 $\:\mu\:$g/L and external loading thresholds for the federally-recognized TMDL are 486, 1451, 799, 349 tons per year for OTB, HB, MTB, and LTB, respectively (Fig. 2) (Janicki & Wade, 1996; Janicki et al., 2000; Tampa Bay Nitrogen Management Consortium, 2010).

Salinity changes from present to fifty years in the future were estimated using the slopes from linear regressions from 1975 to present for the annual average salinities in each bay segment, i.e., to obtain estimated change in ppth per year. This approach assumed a slight but constant decrease in salinity over the next few decades, though it does not explicitly consider how accelerating sea level rise may counteract these changes. However, the estimated changes per year were in agreement with past estimates (i.e., Beck et al., 2024) and may include the combined effects of rainfall changes and sea level rise given that both have been occurring for the prior period of record. Similar loading scenarios, as above, were also assessed - current load, loading at the TMDL threshold, half the threshold, and twice the threshold. The simulations and scenarios were applied to the 2012–2024 period of record for each bay segment. Changes in salinity were implemented for the chosen time period at one year time steps up to “50 years” using the expected change per year, where the observed salinity time series was multiplied by the slope and the number of years in the “future” (one to 50).

Similarly, loading scenarios were evaluated for the four different conditions described above using the observed loading or scaled accordingly within each year, such that the sum of monthly loadings each year was equal to the annual sum for each relative scenario (i.e., half the TMDL, equal to TMDL, or double the TMDL). Although long-term reductions in loading have been observed over several decades, evaluating upper and lower bounds of potential loads relative to the TMDL threshold for each bay segment provides a more contextualized assessment. While we recognize this does not represent a realistic scenario where loading is consistent fifty years into the future, the effects of gradual salinity changes can be more readily evaluated for fixed constants of loading. Moreover, loadings have been relatively consistent since 2012 (the simulation period, Table S8) and emphasizing that the “status quo” may not remain protective in the future is also a worthwhile assessment. Practically, the results can be interpreted as the relative effects of potential salinity and loading changes if all other conditions within the chosen year period persisted for fifty years into the future.

## Results

### Model Performance

**Fig. 3:**
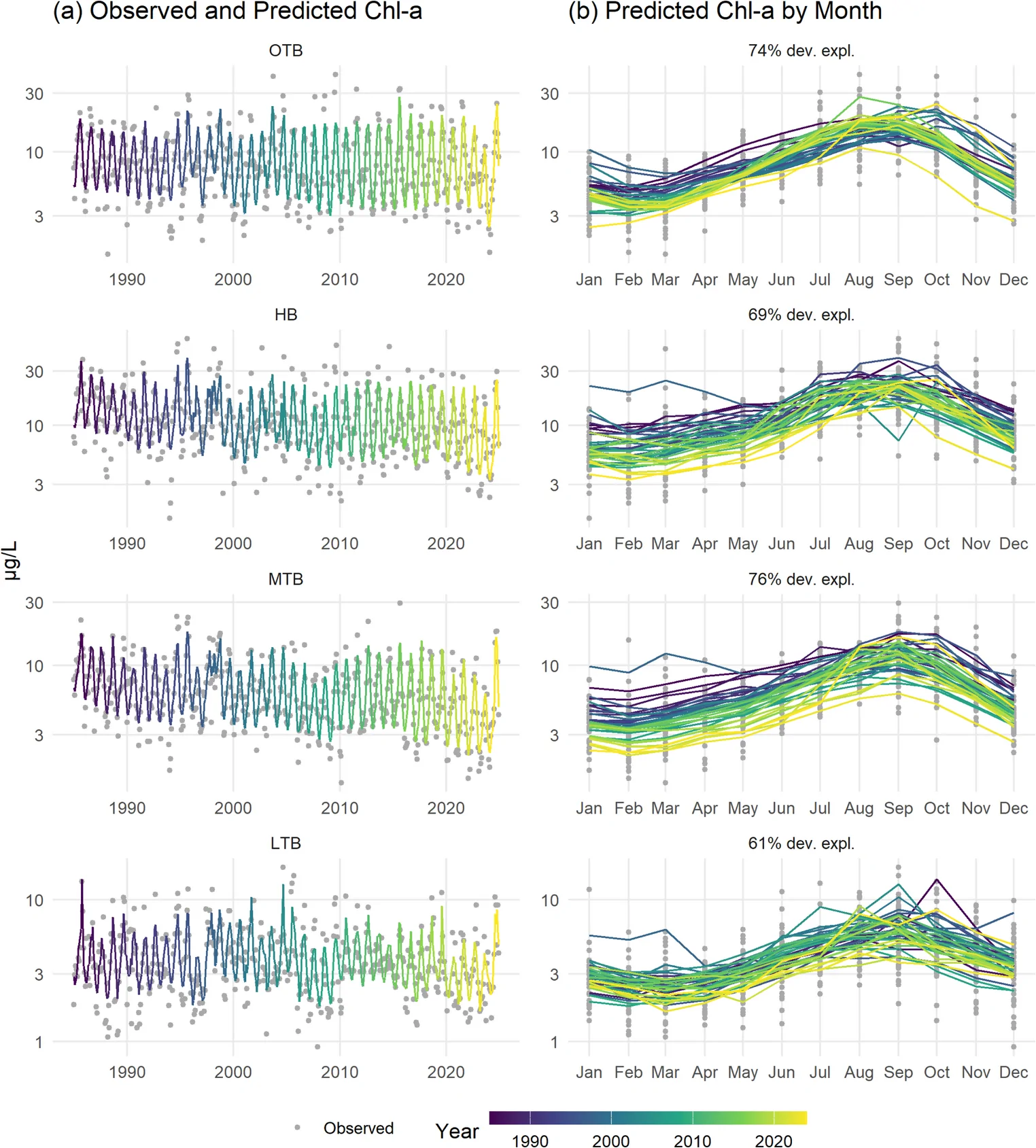
Observed and predicted chlorophyll-a (μg/L) for the four major bay segments of Tampa Bay. Observed and predicted values are shown continuously over time (**a**) and by month (**b**). Color shading indicates year. Rows are arranged by bay segment with the top labels in the left column showing the bay segment and the right column showing deviance explained

All bay segments had overall explained deviance near 70% (OTB 74%, HB 69%, MTB 76%), except LTB, which had the lowest at 61% (Fig. 3; Table 1). Model predictions over time captured the seasonality of chlorophyll-a and substantial inter-annual variability in the seasonality. The long-term decreases in chlorophyll-a were notable in the upper bay segments, i.e., purple lines earlier in the time series had higher chlorophyll-a predictions than the green and yellow lines later in the time series for OTB and HB (Fig. 3b). All bay segments had individual smoothers for decimal time, day of year, and salinity that explained a unique portion of the variance in chlorophyll-a (Table 1, Figure S2), although the defined relationships varied from linear (estimated degrees of freedom, edf = 1; e.g., decimal time for OTB, salinity for OTB and HB) to highly non-linear (edf > > 1; day of year for all bay segments). None of the bay segments had individual smoothers for nitrogen loading that described a unique portion of the explained deviance in the models (Table 1). However, every bay segment except MTB had at least one interaction smoother with nitrogen loading that described a unique amount of the deviance, suggesting an effect of loading that was dependent on values of other predictors (loading and decimal time for OTB and HB; loading and salinity for LTB, Table 1, Figure S3).

**Table 1:** Generalized Additive Model (GAM) summary statistics for chlorophyll-a predictions by bay segment in Tampa Bay (n = 480 for each). Results for the overall model fit are shown with the Generalized Cross-Validation (GCV) score and deviance explained (Dev. Expl.). Summary statistics for the smoothers used in each model are also shown with the estimated degrees of freedom (edf), reference degrees of freedom (Ref.df), F-statistic (F), and p-value (p, bold < α = 0.05). s() indicates a single spline smooth for the predictor and ti() indicates a tensor product smoother interaction between two predictors. dec_time: decimal time; doy: day of year; sal: salinity (ppth); tn_load: total nitrogen load (tons/month). OTB: Old Tampa Bay; HB: Hillsborough Bay; MTB: Middle Tampa Bay; LTB: Lower Tampa Bay

Bay Segment	GCV	Dev. Expl.	Smoother	edf	Ref.df	F	p
OTB	1159	0.74	s(dec_time)	1	1	10.01	**0.002**
			s(doy)	5.96	8	50.08	**< 0.001**
			s(sal)	1	1	13.03	**< 0.001**
			s(tn_load)	1.91	2.45	1.49	0.251
			ti(dec_time,doy)	7.11	12	2.05	**< 0.001**
			ti(dec_time,sal)	8.89	10.96	2.91	**< 0.001**
			ti(sal,doy)	3.57	12	1.8	**< 0.001**
			ti(dec_time,tn_load)	1	1.01	4.55	**0.033**
			ti(tn_load,doy)	0	12	0	0.501
			ti(tn_load,sal)	1.84	2.24	0.89	0.460
HB	1347	0.69	s(dec_time)	3.87	4.8	21.02	**< 0.001**
			s(doy)	5.24	8	28.07	**< 0.001**
			s(sal)	1	1	41.25	**< 0.001**
			s(tn_load)	2.69	3.45	2.2	0.079
			ti(dec_time,doy)	0.78	12	0.08	0.264
			ti(dec_time,sal)	7.23	9	1.81	0.064
			ti(sal,doy)	3.35	12	0.69	**0.019**
			ti(dec_time,tn_load)	2.47	2.89	2.93	**0.041**
			ti(tn_load,doy)	0	12	0	0.439
			ti(tn_load,sal)	2.23	2.67	2.49	0.114
MTB	970	0.76	s(dec_time)	11.09	13.76	15.17	**< 0.001**
			s(doy)	5.76	8	47.51	**< 0.001**
			s(sal)	2.75	3.48	24.21	**< 0.001**
			s(tn_load)	1.26	1.47	1.59	0.136
			ti(dec_time,doy)	1.58	12	0.42	**0.028**
			ti(dec_time,sal)	6.51	8.3	1.52	0.144
			ti(sal,doy)	1.88	12	0.45	**0.031**
			ti(dec_time,tn_load)	1	1	0.03	0.858
			ti(tn_load,doy)	0	12	0	0.403
			ti(tn_load,sal)	1	1	3.75	0.053
LTB	836	0.61	s(dec_time)	23.05	27.88	3.72	**< 0.001**
			s(doy)	4.73	8	23.71	**< 0.001**
			s(sal)	2.52	3.22	13.36	**< 0.001**
			s(tn_load)	1	1	3.24	0.073
			ti(dec_time,doy)	4.42	12	0.68	0.050
			ti(dec_time,sal)	1.83	2.28	0.41	0.623
			ti(sal,doy)	0	12	0	0.883
			ti(dec_time,tn_load)	1	1	0.15	0.702
			ti(tn_load,doy)	1.51	12	0.29	0.063
			ti(tn_load,sal)	4.6	5.99	2.26	**0.038**

Differences in correlations between observed and predicted chlorophyll-a were also shown by annual groupings and season (Table 2), suggesting fit varied by time in each bay segment. All annual groupings by bay segment had correlations greater than 0.7. The highest annual grouping correlation was observed for 2012 to 2024 for OTB ($\:\rho\:$ = 0.90), whereas the lowest was observed for 1999 to 2011 for LTB ($\:\rho\:$ = 0.74). By season, the dry season had generally higher correlations than the wet season across the annual groupings and bay segments (mean $\:\rho\:$ = 0.78, 0.68, respectively). The highest seasonal correlation was observed for the dry season from 2012 to 2024 in OTB ($\:\rho\:$ = 0.92), whereas the lowest correlation was observed for the wet season from 1999 to 2011 in OTB ($\:\rho\:$ = 0.54). Only one instance of the wet season correlation being greater than the dry season correlation was observed, which occurred for 2012–2024 in LTB ($\:\rho\:$ = 0.81, 0.62, respectively).

**Table 2:**
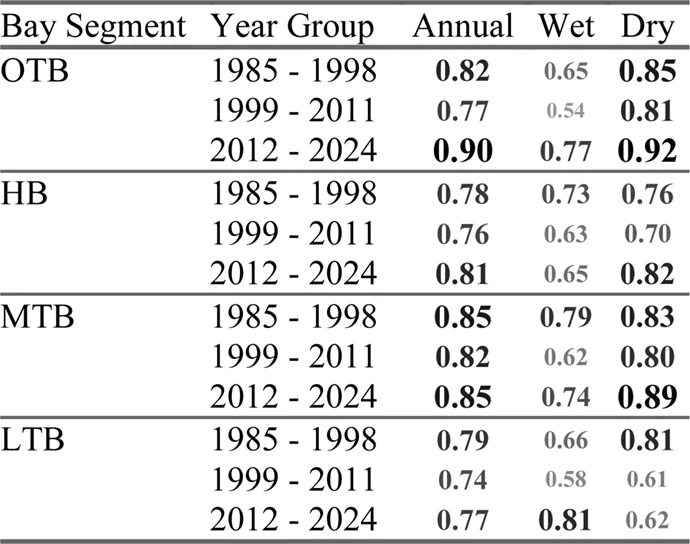
Correlations between monthly observed and predicted chlorophyll-a for different annual and seasonal groupings for each bay segment. The Annual column shows the correlation for the entire year grouping (1985-1998, 1999-2011, or 2012-2024) and the remaining columns show the seasonal correlations within the year group. Font shading and size are proportional to the strength of the correlations. See Table 1 for individual model summary statistics. Wet: June to September; Dry: October to May. OTB: Old Tampa Bay; HB: Hillsborough Bay; MTB: Middle Tampa Bay; LTB: Lower Tampa Bay

### Drivers of Chlorophyll-a Change

**Fig. 4:**
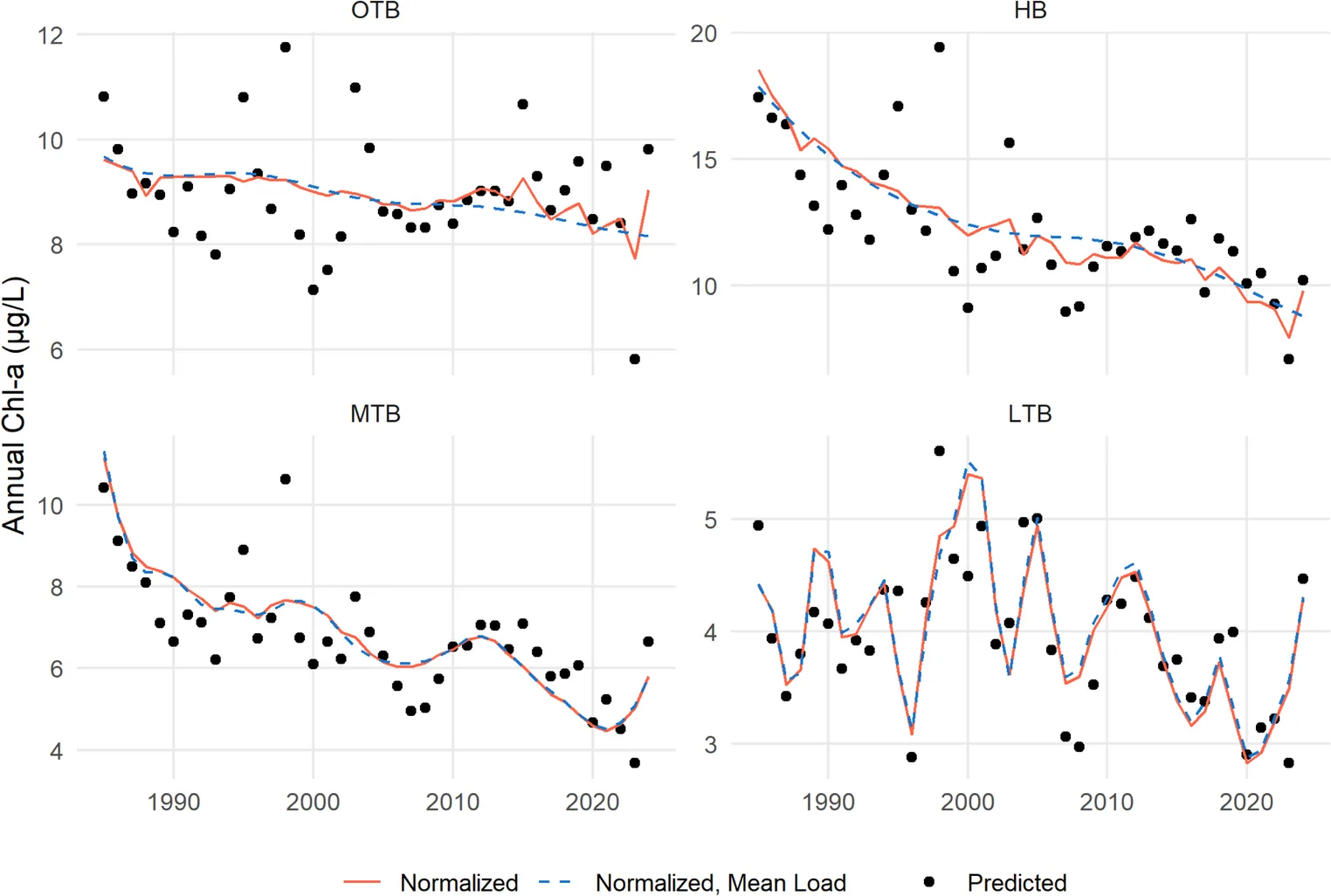
Annual predictions, normalized estimates, and normalized estimates at mean loading for chlorophyll-a by major bay segment from the separate GAMs. Normalized values (solid lines) are those that remove the salinity influence using the mean result of predictions across a range of salinity values at each time step, whereas the normalized, mean load values (dashed lines) are salinity-normalized values at a constant mean load for each bay segment. OTB: Old Tampa Bay; HB: Hillsborough Bay; MTB: Middle Tampa Bay; LTB: Lower Tampa Bay

Annually-averaged model predictions showing the salinity-normalized and salinity-normalized results at mean loading provided a general description of the differing effects of salinity and loading on chlorophyll-a over time (Fig. 4). For each bay segment, the plots were evaluated based on differences between the predictions (black points), salinity-normalized results (solid red lines), and salinity-normalized results at mean loading (dashed blue lines). All bay segments except LTB had normalized results (salinity and salinity at mean loading) that differed largely from model predictions, suggesting that salinity and/or loading were key drivers of chlorophyll-a that varied over time. For LTB, the normalized results generally followed the predictions, suggesting a relatively lower influence of salinity and loading in the model fit to chlorophyll-a in this bay segment. Further, OTB and HB had salinity-normalized results that differed from the results at mean loading, whereas the results for MTB and LTB had salinity-normalized results that were similar to those at mean loading. This suggested that loading had more unique effects on chlorophyll-a in OTB and HB, whereas a loading effect could not be distinguished from salinity for MTB and LTB. The unique effects of loading in OTB were most notable later in the time series, as indicated by larger deviations of the solid red lines from the dashed blue lines. Comparing MTB and LTB suggests that salinity had a larger influence on chlorophyll-a in MTB and loading could not be distinguished from the other variables in the model for either.

**Fig. 5:**
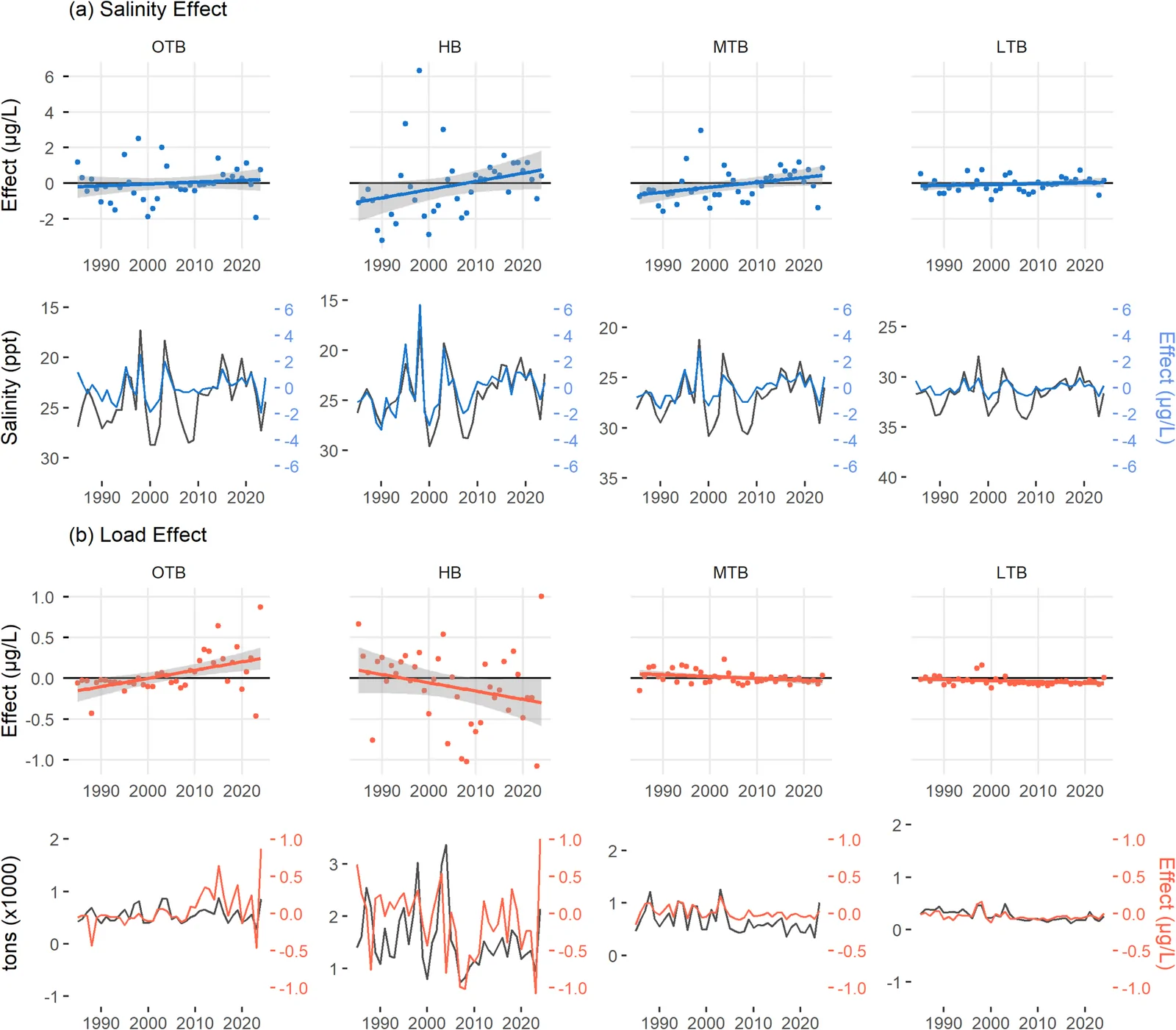
Unique effects of salinity (**a**) and loading (**b**) on chlorophyll-a concentrations over time by bay segment. Points in the first and third rows represent values for a given year where the model predictions minus the salinity-normalized results show the effect of salinity (**a**) and the salinity-normalized results minus the normalized results at mean loading show the effect of loading (**b**). Linear trends and standard errors across years are also shown. Plots in rows two and four show the same values for the salinity or loading effects as in rows one and three on the right axes, but with the actual salinity and loading values included on the left axes. Note that the salinity axis in row two is reversed. OTB: Old Tampa Bay; HB: Hillsborough Bay; MTB: Middle Tampa Bay; LTB: Lower Tampa Bay

The results in Fig. 4 are shown differently in Fig. 5 by taking the differences between the predictions and the salinity-normalized results (salinity effects, Fig. 5a) and the differences between the salinity-normalized results and the salinity-normalized results at mean loading (loading effects, Fig. 5b). The differences in scale between the salinity and loading effects suggest that the former explained more of the variation of chlorophyll-a. Linear trends by bay segment across the period of record also demonstrated that salinity effects through gradual freshening over time have contributed to higher chlorophyll-a concentrations in all bay segments, with the effect decreasing toward the mouth of the bay. However, loading effects differed by bay segment, where the effects of lower loading over time have contributed to lower chlorophyll-a in all bay segments, except OTB, where a slight increase in load over time means an opposite chlorophyll-a trend was observed. The effects of salinity and loading on chlorophyll-a closely followed actual trends in salinity and loading (Fig. 5, rows two and four).

The relative differences in the predictions, salinity-normalized results, and salinity-normalized results at mean loading were further evaluated by annual groupings and season (Table 3). The results are expressed as mean differences in chlorophyll-a for the predictions in Fig. 4 to describe the magnitude and direction of the effects of salinity (predictions minus salinity-normalized results) and loading (salinity-normalized results minus salinity-normalized results at mean loading) in the left and right sides of Table 3, respectively. As described above, chlorophyll-a concentrations were higher over time from salinity effects (freshening), particularly during the wet season in HB and MTB. Similarly, chlorophyll-a concentrations increased over time from loading effects, although the effects were generally lower than for salinity and varied by season. The largest increases from loading by season, particularly in OTB and HB, were observed for the wet season where the effect increased across year groupings, whereas the dry season showed the opposite trend. Overall, these results provide greater insight into the annual changes by exploring times of year when the changes may be most dramatic (or even counter to one another).

**Table 3:**
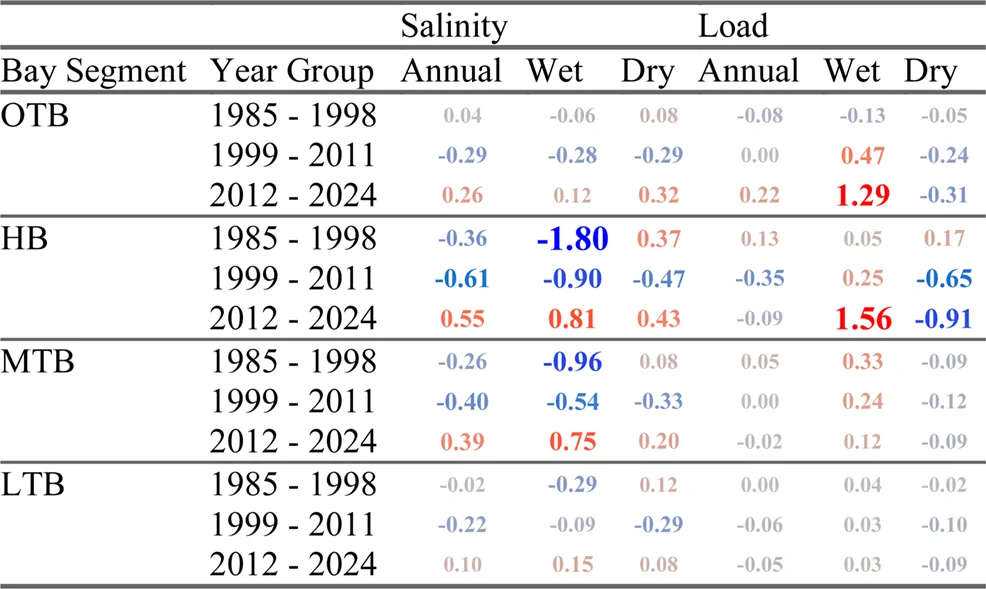
Mean differences (μg/L) for monthly predictions minus salinity-normalized results (Salinity) and salinity-normalized results minus salinity-normalized results at mean loadings (Load). The values in the salinity columns can be considered the relative effects of changes in salinity on chlorophyll-a, whereas those in the load columns are relative effects of loading on chlorophyll-a independent of salinity. The Annual columns show the differences for the entire year grouping (1985-1998, 1999-2011, or 2012-2024) and the remaining columns show the differences by season within the year group. Font size is proportional to the magnitude of the effects, whereas colors show negative (blue) or positive (red) effects on chlorophyll-a. Wet: June to September; Dry: October to May. OTB: Old Tampa Bay; HB: Hillsborough Bay; MTB: Middle Tampa Bay; LTB: Lower Tampa Bay

**Fig. 6:**
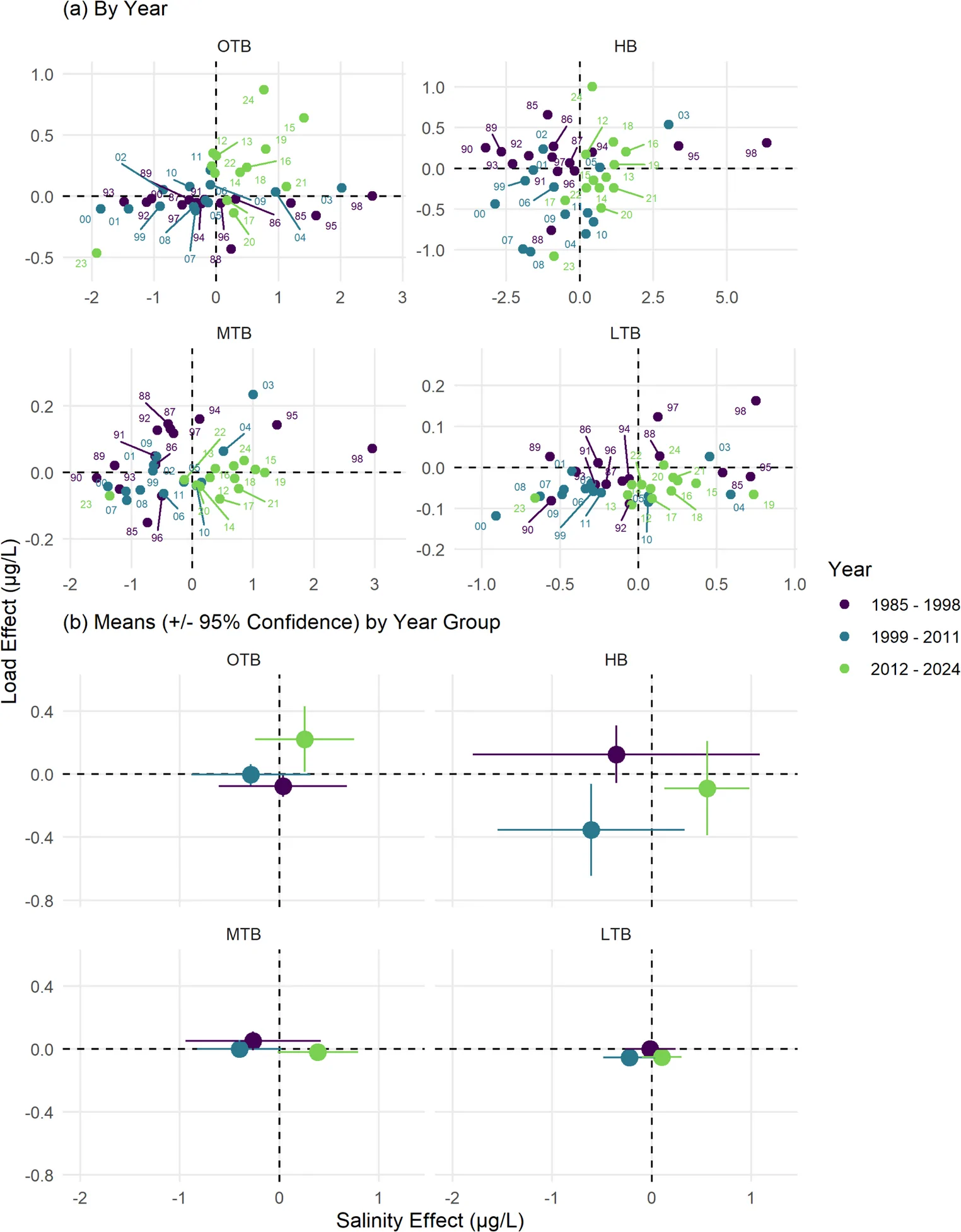
State-space representations of modelled annual chlorophyll-a response to salinity (x-axis) or nitrogen load (y-axis). Points represent values for a given year in (**a**). Means and 95% confidence intervals for the year groups are shown in (**b**). The axis scales are not fixed in (**a**) to better visualize inter-annual changes within each bay segment, whereas the scales are fixed in (**b**) to emphasize differences in the magnitude of the drivers between bay segments. OTB: Old Tampa Bay; HB: Hillsborough Bay; MTB: Middle Tampa Bay; LTB: Lower Tampa Bay

The state-space representation provided a complementary interpretation of the effects of salinity and loading on chlorophyll-a over time (Fig. 6, these are the same results as in Fig. 5 but plotted in both dimensions). Overall by annual grouping (1985–1998, 1999–2011, and 2012–2024), OTB suggested a shift towards higher chlorophyll-a from both load and salinity influences in 2012–2024 as shown by a shift in the mean effects towards the upper right of the plot (Fig. 6b). A shift towards the right on the x-axis for the 2012–2024 grouping for all bay segments suggested higher chlorophyll-a values influenced by fresher salinities. Although general trends by annual groupings were shown by the mean effects in Fig. 6b, substantial inter-annual variability was shown for some years (Fig. 6a). For example, exceptionally wet years that had a disproportionate effect on chlorophyll-a were seen as outliers in 1998 on the x-axis for OTB, HB, and MTB, corroborating El Niño rainfall driven impacts that have previously been reported (Greening et al., 2014). Similarly, years with higher than normal chlorophyll-a concentrations from loading effects were seen in 2024 as high values on the y-axis for OTB and HB.

### Likelihood of Exceeding Regulatory Criteria

Scenario assessments for recent years (2012–2024) suggested future reductions in salinity will contribute to increased likelihoods of exceeding the respective chlorophyll-a thresholds for each bay segment (Table 4). However, these effects were trivial for all bay segments except OTB (described below) because thresholds have not been exceeded in recent years for the other segments. That is, future reductions in salinity in HB, MTB, and LTB produced slight increases in the likelihood of exceedance, though overall likelihoods were comparatively minimal (still < 10% likelihood over 50 years). One exception is HB under a doubled TMDL scenario, such that the likelihood of exceeding the chlorophyll-a regulatory threshold rose from ~ 1% at current load conditions to 15% at present and 19% in fifty years with reduced salinity. These increased likelihoods are still marginal compared to OTB.

**Table 4:** Likelihoods of exceeding regulatory chlorophyll-a thresholds for each bay segment for different loading scenarios under projected future salinity changes. Results shown are likelihoods of exceeding the threshold from 10,000 simulations from each model and future year (1 to 50) for the 2012-2024 time period. Standard deviations and the relative changes from present for years 25 and 50 are shown in parentheses. OTB: Old Tampa Bay; HB: Hillsborough Bay; MTB: Middle Tampa Bay; LTB: Lower Tampa Bay

				TMDL Load Factor		
BaySegment	Time	SalinityChange (ppth)	Actual Load	0.5	1	2
OTB	Present	—	39.9 (10.2)	20 (8.7)	34.8 (10.3)	56.3 (10.1)
	Year 25	-0.95	47.6 (9.9, ↑7.7)	27.3 (9.7, ↑7.3)	43 (10.2, ↑8.3)	62.9 (10, ↑6.5)
	Year 50	-1.89	55.6 (9.7, ↑15.7)	36.3 (10, ↑16.2)	52 (10.1, ↑17.3)	69.4 (9.1, ↑13)
HB	Present	—	1.3 (2.6)	0 (0.5)	1.2 (2.5)	14.5 (7.6)
	Year 25	-0.66	1.6 (2.8, ↑0.3)	0 (0.5, ≈0)	1.5 (2.8, ↑0.3)	16.9 (8.1, ↑2.4)
	Year 50	-1.33	2 (3.1, ↑0.6)	0.1 (0.6, ≈0)	1.8 (3.1, ↑0.6)	18.9 (8.3, ↑4.3)
MTB	Present	—	0.6 (1.7)	0.7 (2)	0.7 (1.9)	0.9 (2.1)
	Year 25	-0.58	1.2 (2.4, ↑0.6)	1.6 (2.8, ↑0.9)	1.4 (2.7, ↑0.7)	1.4 (2.6, ↑0.5)
	Year 50	-1.17	2.4 (3.5, ↑1.9)	3.5 (4.1, ↑2.7)	2.6 (3.6, ↑1.9)	2.2 (3.2, ↑1.3)
LTB	Present	—	2.1 (3.2)	1.9 (3)	3.7 (4)	5.3 (4.8)
	Year 25	-0.76	4.6 (4.4, ↑2.5)	4.3 (4.3, ↑2.4)	6.8 (5, ↑3.1)	8.6 (5.7, ↑3.3)
	Year 50	-1.51	9 (5.7, ↑6.9)	8.7 (5.8, ↑6.8)	11.1 (6, ↑7.4)	13.4 (6.7, ↑8.2)

**Fig. 7:**
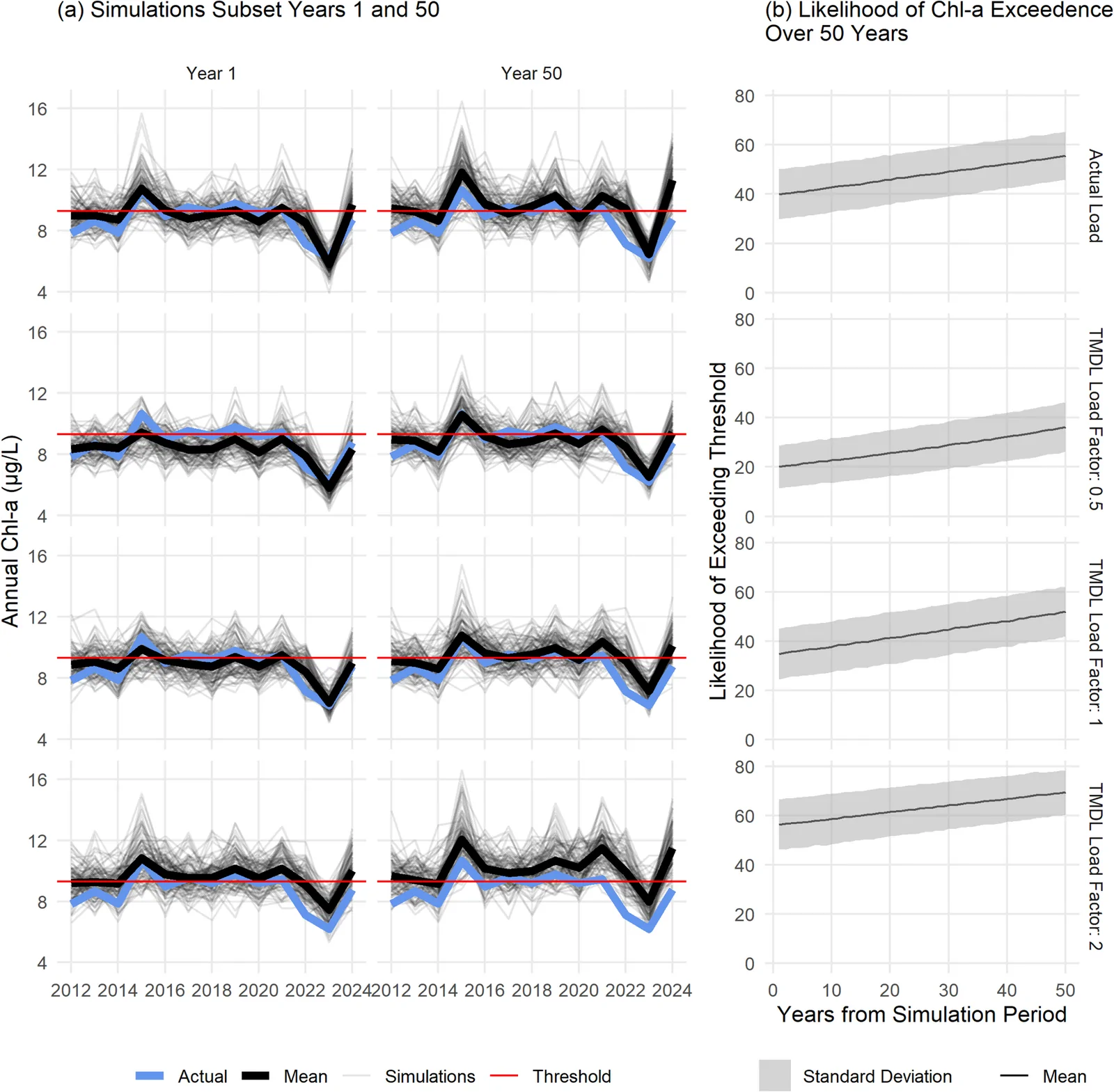
Estimated chlorophyll-a for a subset of 100 simulations (**a**) and likelihood of exceeding regulatory thresholds with 10,000 simulations (**b**) in OTB under different loading conditions and salinity changes. The left column in (**a**) shows simulated chl-a for the first year where salinity is set at observed values and the right column shows results for expected salinity fifty years from present for the time period from 2012–2024. Nitrogen load values were set to the actual observed load (top row), half the TMDL (second row), the TMDL (third row), and double the TMDL (bottom row). Lines in (**b**) show the average and standard deviations of likelihoods of exceeding the thresholds over time for 10,000 simulations using the OTB model

The remainder of this section focuses on the likelihood that OTB will exceed regulatory criteria under projected salinity changes and different loading scenarios. Because actual regulatory exceedances of chlorophyll-a were observed from 2012 to 2024 (five years in this period), the likelihood at present under observed salinity and loading conditions was approximately 40% (Table 4). Projected changes in salinity and varying load scenarios had more impact on the overall likelihood of attaining chlorophyll-a regulatory standards. As an example, a subset of 100 simulations showed the effects of changing salinity for each loading scenario on predicted annual chlorophyll-a relative to the regulatory criteria of 9.8 $\:\mu\:$g/L (Fig. 7a). The left column in Fig. 7a shows the results for present salinity and the right column shows the results for salinity fifty years from present (at a decrease of 0.04 ppth per year), where the rows (from top to bottom) show results for the actual load, half the TMDL loading, TMDL loading, and twice the TMDL loading. The solid black lines show the average chlorophyll-a across the simulations, relative to the actual chlorophyll-a (solid blue lines) for each scenario. The decrease in salinity resulted in higher predicted chlorophyll-a for all loading scenarios. As expected, higher loading conditions also resulted in higher chlorophyll-a.

**Fig. 8:**
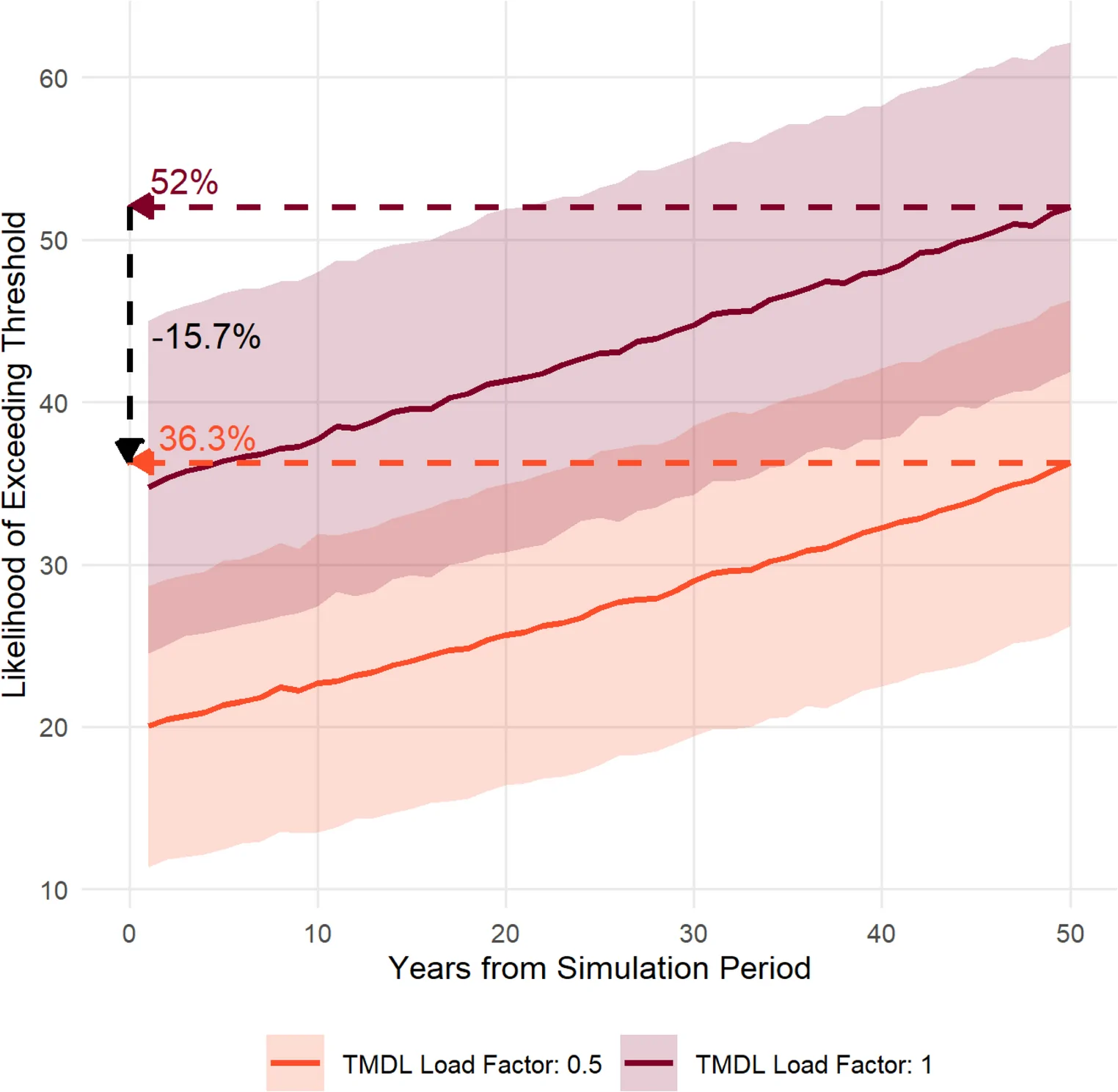
Likelihood of exceeding regulatory thresholds in OTB over fifty years if nutrient loading is maintained at the current TMDL or at half the TMDL. Solid lines show the average and standard deviations of likelihoods of exceeding the thresholds over time for 10,000 simulations using the OTB model. Dashed lines show the estimated likelihood at year 50 and the difference between the two scenarios

Results across all years (1 year to 50) for the full 10,000 simulations show the overall likelihood of exceeding the regulatory threshold in OTB under the different scenarios (Fig. 7b). If current loadings are maintained, the likelihood of exceeding the regulatory thresholds increases 16% by year 50 to 56% (Table 4). Relative increases in the likelihood are similar for each loading scenario, although the curves shift up or down proportionally depending on the loading. Understandably, reducing the load by half shifted the likelihood curves down, whereas doubling the load shifted the curves higher. Importantly, maintaining loading at the TMDL (slightly lower than the observed average loading from 2012 to 2024) showed that the likelihood of exceeding the regulatory criteria increases from 35% to 52% by year 50 with expected changes in salinity. If loading were reduced to half the TMDL, the likelihood of exceeding the regulatory thresholds decreased by 16% to 36% by year 50 (Fig. 8), suggesting that load reductions beyond the TMDL may be needed to maintain an acceptable likelihood of meeting regulatory chlorophyll-a thresholds in the future.

## Discussion

The underlying management paradigm for Tampa Bay emphasizes the control of external nutrients to create a light environment that is supportive of seagrass. This paradigm has effectively supported the long-term recovery of seagrass in Tampa Bay, whereas shifting baselines, both present and in the future, may diminish the effectiveness of this approach (Sherwood et al., 2017; Beck et al., 2024). The management paradigm for Tampa Bay was conceived over thirty years prior to this work based on conditions of the bay in the 1990s (Janicki & Wade, 1996; Janicki et al., 2000). Tampa Bay’s ecology has changed since this time and the primary objective of this work was to assess the time-varying effects of loading and salinity on the bay’s water quality. Our results demonstrated that chlorophyll-a conditions are affected by both, particularly in the upper bay, and that the dominant drivers have shifted over time. This information has ecological importance by demonstrating how shifting baselines will continue to alter key drivers of water quality that can influence the ecosystem as a whole. This information also has regulatory and management implications. Mitigating external nutrient loads has been a fundamental approach towards estuarine restoration, whereas baywide salinity controls typically are not. In anticipation of future salinity conditions, additional load reductions beyond protective levels identified under previous paradigms may be warranted, particularly in OTB.

The simulation results suggested an increased likelihood of exceeding regulatory thresholds with projected future decreases in salinity. These analyses also underscored that nutrient loads remain a crucial lever for managers to affect water quality outcomes. In OTB, where chlorophyll-a regulatory exceedances have occurred in several past years, changing salinity conditions may require further load reductions beyond the federally-established TMDL to maintain water quality supportive of seagrass into the future. Even if the nutrient TMDL is maintained (it is currently exceeded by 6 tons for the last assessment period based on a five-year average), the likelihood that the chlorophyll-a threshold will be exceeded increases by 8% in 25 years and by 17% in fifty years to an overall likelihood of 52%. These increases must also be considered relative to potential future loading trends if insufficient actions are taken to mitigate sources or the effects are compounded by rainfall-driven salinity changes we forecasted herein. Although total loads across all sources in OTB have been relatively stable since 1985 (with substantial inter-annual variability, Fig. 2c), contributions from non-point sources (stormwater primarily) have increased at a rate of 2.6 tons per year. These load increases, if not sufficiently addressed, will further increase the likelihood of exceeding regulatory criteria in the future. The simulation models suggest that current loadings, particularly in OTB, will need to be substantially reduced from existing TMDL levels to keep the likelihood of exceeding the chlorophyll-a regulatory criteria below 50% through the next half-century.

Model predictions compared to salinity-normalized and salinity-normalized results at mean loading suggested that chlorophyll-a in (1) OTB and HB were influenced by both salinity and loading, (2) MTB was influenced primarily by salinity, and (3) LTB could not be distinguished as influenced by either. These relationships were also variable over time, particularly in OTB, where pure loading effects appear to have recently increased in importance. This result has management implications where exceedances of the chlorophyll-a regulatory threshold have been observed in recent years. Current regulatory compliance assessments for the nutrient loadings in each bay segment were established as nitrogen and phosphorus delivery ratios, where total loads are scaled based on total hydrologic inputs to a bay segment (Tampa Bay Nitrogen Management Consortium, 2010). This allows flexibility during wet years when loading is higher than normal so that stormwater managers are not unnecessarily penalized. For all bay segments except OTB, there has been agreement between meeting the chlorophyll-a threshold and the nutrient load ratios. Recent and frequent exceedances of the chlorophyll-a threshold in OTB, while consistently attaining nutrient loading ratios, have presented a mismatch for compliance assessment and delayed actions by bay managers. Because the nutrient loading ratios are based on hydrologic conditions from a 1992–1994 reference period, changing hydrology and rainfall patterns suggest this approach no longer describes a meaningful ecological relationship (Beck et al., 2024; Wang et al., 2026). Chlorophyll-a attainment based on absolute loadings would be a more meaningful assessment, at least for OTB.

The model results were interpreted as the unique effects of both nutrient loading and salinity, one or the other, or neither on chlorophyll-a. Although loading was suggested as having less of an influence on chlorophyll-a in MTB and LTB, the results do not imply external loading is unimportant in these segments. Rather, the results must be qualitatively interpreted between bay segments such that loading is considered a more influential driver of chlorophyll-a in the upper segments, not that it is wholly unimportant for the ecology of the lower bay segments. Further, the GAM structure was specifically selected to isolate the effects of key drivers and is used herein as a relatively simplistic interpretation in comparison to more complex mechanistic approaches that simulate chlorophyll-a. Controlling external nitrogen loads to each bay segment remains a key focus for management efforts to maintain or improve water quality supportive of seagrass in Tampa Bay.

Recognizing how salinity influences or is a direct proxy for causal mechanisms of chlorophyll-a dynamics in each bay segment is critical for the interpretation of the results. Salinity is not independent of loading, as nutrient inputs to the bay are largely dependent on annual rainfall. Extreme wet or dry years have coincided with high and low nutrient loading years, which are prominent as the annual outliers in Figs. 5 and 6. From a statistical perspective, loading and salinity are correlated but not so excessively as to prevent both from being used as unique predictors in the models (unique signals from both were identified). Given their varying importance in the models for each bay segment, salinity can be interpreted as having a unique effect on chlorophyll-a independent of loading. The most likely interpretation is that salinity is a proxy for phytoplankton ecology, flushing, residence time, and tidal exchange (e.g., Hagy et al., 2000; Meyers et al., 2015). Although lower salinity likely reflects greater freshwater inputs, the association with residence time may be more complicated. In many estuaries, higher freshwater inputs may increase flushing, reduce residence time, and export more nutrients and phytoplankton biomass seaward (e.g., Sin et al., 1999; Lu & Gan, 2015). This may not be the case in Tampa Bay, particularly for OTB, given the association of higher chlorophyll-a with more fresh conditions. Rather, periods of lower salinity generally create conditions where the combination of loads from stormwater inputs and lack of exchange with the Gulf promotes an environment conducive for phytoplankton growth. Such periods of time could be visually interpreted from Fig. 4 when the model predictions are much higher than the solid red lines showing the salinity-normalized values. The opposite could be true during periods of high salinity (i.e., predictions well below the solid red lines). This is particularly evident when comparing 2023 and 2024 in Fig. 4. In 2023, a watershed-wide drought affecting all bay segments appeared to impart more tidal controls on salinities and resulted in lower chlorophyll-a predictions. In 2024, the opposite occurred whereby extreme rainfall from three tropical cyclone events late in the year produced lower than average salinities and higher predicted chlorophyll-a across all the segments.

Our approach offers a relatively novel application of GAMs to describe long-term trends in chlorophyll-a and how drivers have changed. We build on similar past models used for these purposes (Richards et al., 2013; Murphy et al., 2019; Beck et al., 2022), although most of the existing applications have focused on trend description (e.g., how has chlorophyll-a changed? ) and not describing changing causal mechanisms (e.g., why has chlorophyll-a changed? ). GAMs have had far fewer applications in the latter context for long-term monitoring data in estuaries (but see, Murphy et al., 2021; Schramm, 2023). The use of GAMs to simulate chlorophyll-a trends under different scenarios is also novel and highlights a strength of these models for evaluating future conditions or management interventions. Other similar but functionally different methods (e.g., WRTDS models) cannot be readily used for these purposes. Mechanistic models have also traditionally filled these roles, particularly for developing TMDLs in impaired waters (Borsuk et al., 2002; Wool et al., 2003; Borah et al., 2006). While mechanistic models will continue to support TMDL development, GAMs are more easily adapted to support management, either through modification of the core model structure or with more flexible scenario assessments.

Finally, the scenario assessments using simulated results from the GAMs also assumed that current chlorophyll-a regulatory criteria are sufficient and will continue to support a light environment for seagrasses. These chlorophyll-a thresholds, much like the loading thresholds, were developed with data from the 1990s and changes to the bay’s ecology warrant further interrogation. Optical models, in situ light measurements, and current seagrass coverage maps cross-referenced with bathymetry need to be leveraged to determine if current chlorophyll-a thresholds are still sufficient to maintain light environments at the deep edge of seagrass for each bay segment (Johansson, 2022). Evaluating (and potentially correcting for) other water quality constituents that attenuate light, such as color and turbidity, in concert with chlorophyll-a will be important considerations (Chen et al. 2007a, b; Le et al. 2013). Although there are no complementary standards for water quality parameters other than chlorophyll-a in Tampa Bay that have been developed to support the seagrass light environment, evaluating alternative drivers of light attenuation will help develop expectations of times when seagrasses may still be vulnerable. For example, high rainfall events, such as during tropical storms, can elevate dissolved organic carbon that alters water color but not chlorophyll-a (Avery et al. 2004; Chen et al. 2007a, b). The effects of these events on light attenuation and long-term seagrass ecology are not well understood in Tampa Bay.

### Conclusions

This study evaluated the competing effects of nutrient loading and salinity on chlorophyll-a dynamics in the four major bay segments of Tampa Bay. Dominant drivers have changed over time, particularly for the upper bay segments (OTB, HB), which has relevance when considering that current management paradigms for the bay were conceived over thirty years ago when conditions were much different than those at present. We further demonstrated how future salinity changes, which are difficult if not impossible to manage, will lead to higher likelihoods of exceeding regulatory criteria in the future, such that current loading thresholds may be insufficient to protect water quality. Specifically, nutrient loads in Old Tampa Bay may need to be substantially reduced below the current TMDL to maintain a sufficiently low likelihood of exceeding chlorophyll-a regulatory standards. These models were developed only in the context of nutrient loading and salinity, and it is recognized that additional system-wide changes may further stress conditions in the bay. In particular, increased temperatures from climate change will not only physically stress the bay’s resources (e.g., seagrass growth and mortality, Beck et al., 2024) but will also alter physiological response of phytoplankton and nutrient cycling rates (Lassen et al., 2010; Statham, 2012). Additional climate change stressors (e.g., sea level rise, Alarcon et al., 2024) and climate variation (e.g., ENSO-related changes in rainfall, Greening et al., 2014) should also be evaluated for their individual or synergistic (e.g., further alteration of salinity) effects on the bay’s ecology. Outcomes from this additional work, when taken with the results herein, can better guide management and protection of critical resources impacted by eutrophication. Our approach can also be leveraged in other estuarine systems where the effectiveness of conventional management paradigms should be assessed relative to changing baselines. 

## Supplementary Information

Below is the link to the electronic supplementary material.

Supplementary Material 1

## Data Availability

All data and analysis code for this manuscript is available on GitHub at https://github.com/tbep-tech/chlhydro-manu.
