# Supplementary material for: Managing the Unmanageable: Understanding the Competing Effects of Salinity and Nutrient Loading on Estuarine Water Quality
Source: Estuaries Coast. 2026 Aug 4;49(6):178. doi: 10.1007/s12237-026-01792-5 (PMC13437595; doi:10.1007/s12237-026-01792-5)
Supplement: Supplementary file 1 [file 12237_2026_1792_MOESM1_ESM.docx]

*Supplement* Managing the unmanageable: Understanding the competing effects of salinity and nutrient loading on estuarine water quality

Marcus W Beck^1,✉^ ([0000-0002-4996-0059](https://orcid.org/0000-0002-4996-0059)), Edward T Sherwood^1^ ([0000-0001-5330-302X](https://orcid.org/0000-0001-5330-302X)), Rebecca R Murphy^2^ ([0000-0003-3391-1823](https://orcid.org/0000-0003-3391-1823)), Kerry Flaherty-Walia^1^ ([0000-0002-6032-0570](https://orcid.org/0000-0002-6032-0570)), Qian Zhang^2^ ([0000-0003-0500-5655](https://orcid.org/0000-0003-0500-5655)), Blake A Simmons^1^ ([0000-0002-1918-3463](https://orcid.org/0000-0002-1918-3463)), and Maya C Burke^1^ ([0000-0002-3847-6501](https://orcid.org/0000-0002-3847-6501))

^1^ Tampa Bay Estuary Program, 263 13th Ave S., Suite 350, St. Petersburg, Florida 33701 USA
^2^ University of Maryland Center for Environmental Science/U.S. Environmental Protection Agency Chesapeake Bay Program, 1750 Forest Drive, Annapolis, Maryland 21401 USA

^✉^ Correspondence: [Marcus W Beck <mbeck@tbep.org>](mailto:mbeck@tbep.org)

## Figures

Fig. S1: Examples demonstrating the salinity normalization method (a), the salinity normalization method at mean loading (b), and the final results (c). Results are obtained by predicting chlorophyll-a concentrations from a fitted GAM across a range of salinities and estimating the average (a) or doing the same but using mean loading (b). The bottom plot (c) shows the normalized curves where the red line is the mean curve from (a), the blue dashed line is the mean curve from (b), and the points are the actual model predictions. The inset in (c) shows how the normalized results are used to identify the unique effects of salinity and loading.

Fig. S2: Effects plots for individual smoothers from Generalized Additive Models applied to each bay segment. Each plot shows the modeled effects on chlorophyll-a of the smoothers identified on the x-axes. The small vertical bars on the x-axis of each plot show the relative distribution of values for each parameter. Confidence intervals (95%) are also shown for each parameter. OTB: Old Tampa Bay; HB: Hillsborough Bay; MTB: Middle Tampa Bay; LTB: Lower Tampa Bay

Fig. S3: Effects plots for bivariate smoothers from Generalized Additive Models applied to each bay segment. Each plot shows the modeled effects on chlorophyll-a of the smoothers identified on the x- and y-axes. Observed values are shown as points to emphasize the typical ranges for each parameter combination. OTB: Old Tampa Bay; HB: Hillsborough Bay; MTB: Middle Tampa Bay; LTB: Lower Tampa Bay

## Tables

Table S1: Comparison of Generalized Additive Models for chlorophyll-a for each bay segment using full models and fit using the select option. Model fit was assessed using Akaike information criterion (AIC, Akaike, 1974), with the lowest AIC value by segment shown in bold. df: degrees of freedom; Dev. Expl.: deviance explained; OTB: Old Tampa Bay; HB: Hillsborough Bay; MTB: Middle Tampa Bay; LTB: Lower Tampa Bay.

|  | Full | | | Select | | |  |
| --- | --- | --- | --- | --- | --- | --- | --- |
| Bay Segment | df | Dev. Expl. | AIC | df | Dev. Expl. | AIC | ΔAIC |
| OTB | 41.6 | 0.74 | 2,287.3 | 40.5 | 0.74 | **2,284.5** | 2.8 |
| HB | 38.2 | 0.69 | **2,673.2** | 36.8 | 0.68 | 2,675.9 | -2.7 |
| MTB | 42.6 | 0.76 | **1,901.6** | 38.3 | 0.75 | 1,901.8 | -0.2 |
| LTB | 55.9 | 0.61 | **1,608.6** | 51.8 | 0.61 | 1,609.0 | -0.4 |
| ΔAIC = Full AIC - Select AIC | | | | | | | |

Table S2: Comparison of Generalized Additive Models for chlorophyll-a for each bay segment using a Gamma and Gaussian (log-link) error distributions. Model fit was assessed using Akaike information criterion (AIC, Akaike, 1974), with the lowest AIC value by segment shown in bold. df: degrees of freedom; Dev. Expl.: deviance explained; OTB: Old Tampa Bay; HB: Hillsborough Bay; MTB: Middle Tampa Bay; LTB: Lower Tampa Bay.

|  | Gamma | | | Gaussian (log) | | |  |
| --- | --- | --- | --- | --- | --- | --- | --- |
| Bay Segment | df | Dev. Expl. | AIC | df | Dev. Expl. | AIC | ΔAIC |
| OTB | 41.6 | 0.74 | **2,287.3** | 80.9 | 0.79 | 2,505.9 | -218.6 |
| HB | 38.2 | 0.69 | **2,673.2** | 48.9 | 0.69 | 2,956.8 | -283.6 |
| MTB | 42.6 | 0.76 | **1,901.6** | 67.4 | 0.80 | 2,050.2 | -148.6 |
| LTB | 55.9 | 0.61 | **1,608.6** | 65.8 | 0.65 | 1,785.8 | -177.2 |
| ΔAIC = Gamma AIC - Gaussian (log) AIC | | | | | | | |

Table S3: Generalized Additive Model (GAM) summary statistics for chlorophyll-a predictions by bay segment in Tampa Bay (n = 480 for each). Results are shown for models with different total nitrogen loading lags, whether salinity or loading were corrected for seasonality, and constraints on the number of smoothers (K) for time, loading, and salinity (flexible or fixed). Loads were evaluated as cumulative sums for the current month up to a three month lag. Results for the overall model fit are shown with the Generalized Cross-Validation (GCV) scores in the top rows and deviance explained (Dev. Expl.) in the bottom rows. OTB: Old Tampa Bay; HB: Hillsborough Bay; MTB: Middle Tampa Bay; LTB: Lower Tampa Bay.

|  |  |  | No Seasonal Correction | | Seasonal Correction | |
| --- | --- | --- | --- | --- | --- | --- |
| Summary | Bay Segment | TN Load Lag | Flexible K | Fixed K | Flexible K | Fixed K |
| GCV |  |  |  |  |  |  |
|  | OTB | None | 1159 | 1160 | 1162 | 1161 |
|  |  | 1 | 1157 | 1157 | 1162 | 1162 |
|  |  | 2 | 1156 | 1157 | 1162 | 1162 |
|  |  | 3 | 1157 | 1157 | 1161 | 1161 |
|  | HB | None | 1347 | 1348 | 1356 | 1356 |
|  |  | 1 | 1349 | 1349 | 1356 | 1356 |
|  |  | 2 | 1348 | 1349 | 1351 | 1352 |
|  |  | 3 | 1351 | 1352 | 1358 | 1357 |
|  | MTB | None | 970 | 971 | 973 | 973 |
|  |  | 1 | 967 | 968 | 970 | 970 |
|  |  | 2 | 963 | 963 | 965 | 965 |
|  |  | 3 | 960 | 960 | 959 | 959 |
|  | LTB | None | 836 | 840 | 839 | 840 |
|  |  | 1 | 839 | 840 | 836 | 837 |
|  |  | 2 | 838 | 840 | 834 | 839 |
|  |  | 3 | 830 | 832 | 828 | 828 |
| Dev. Expl. |  |  |  |  |  |  |
|  | OTB | None | 0.74 | 0.74 | 0.74 | 0.74 |
|  |  | 1 | 0.74 | 0.74 | 0.74 | 0.74 |
|  |  | 2 | 0.75 | 0.75 | 0.74 | 0.74 |
|  |  | 3 | 0.74 | 0.74 | 0.74 | 0.74 |
|  | HB | None | 0.69 | 0.68 | 0.67 | 0.67 |
|  |  | 1 | 0.69 | 0.69 | 0.69 | 0.69 |
|  |  | 2 | 0.69 | 0.69 | 0.70 | 0.70 |
|  |  | 3 | 0.68 | 0.68 | 0.67 | 0.68 |
|  | MTB | None | 0.76 | 0.75 | 0.76 | 0.75 |
|  |  | 1 | 0.75 | 0.75 | 0.76 | 0.75 |
|  |  | 2 | 0.75 | 0.76 | 0.76 | 0.76 |
|  |  | 3 | 0.76 | 0.76 | 0.77 | 0.77 |
|  | LTB | None | 0.61 | 0.52 | 0.60 | 0.52 |
|  |  | 1 | 0.60 | 0.55 | 0.55 | 0.55 |
|  |  | 2 | 0.59 | 0.55 | 0.56 | 0.53 |
|  |  | 3 | 0.61 | 0.57 | 0.57 | 0.57 |

Table S4: Concurvity estimates for the **Old Tampa Bay** Generalized Additive Models. Values range from 0 to 1 and assess the degree of multicollinearity among predictors. Results are shown for models with different total nitrogen loading lags, whether salinity or loading were corrected for seasonality, and constraints on the number of smoothers (K) for time, loading, and salinity (flexible or fixed). Loads were evaluated as cumulative sums for the current month up to a three month lag. Font size and shading are proportional among cells to emphasize differences.

| Seasonal Correction | K | TN Load Lag | s(dec_time) | s(doy) | s(sal) | s(tn_load) | ti(dec_time,doy) | ti(dec_time,sal) | ti(sal,doy) | ti(dec_time,tn_load) | ti(tn_load,doy) | ti(tn_load,sal) |
| --- | --- | --- | --- | --- | --- | --- | --- | --- | --- | --- | --- | --- |
| No | Flexible | None | **0.87** | **0.67** | **0.97** | **0.97** | **0.57** | **0.58** | **0.62** | **0.60** | **0.78** | **0.66** |
|  |  | 1 | **0.84** | **0.68** | **0.98** | **0.97** | **0.61** | **0.61** | **0.70** | **0.60** | **0.83** | **0.69** |
|  |  | 2 | **0.85** | **0.66** | **0.98** | **0.98** | **0.63** | **0.64** | **0.73** | **0.63** | **0.84** | **0.70** |
|  |  | 3 | **0.87** | **0.65** | **0.99** | **0.99** | **0.63** | **0.69** | **0.74** | **0.72** | **0.83** | **0.78** |
|  | Fixed | None | **0.97** | **0.62** | **0.98** | **0.97** | **0.54** | **0.53** | **0.59** | **0.61** | **0.79** | **0.68** |
|  |  | 1 | **0.90** | **0.63** | **0.98** | **0.97** | **0.59** | **0.56** | **0.66** | **0.59** | **0.84** | **0.69** |
|  |  | 2 | **0.88** | **0.61** | **0.98** | **0.98** | **0.60** | **0.60** | **0.70** | **0.63** | **0.84** | **0.70** |
|  |  | 3 | **0.87** | **0.62** | **0.98** | **0.99** | **0.63** | **0.65** | **0.72** | **0.70** | **0.84** | **0.77** |
| Yes | Flexible | None | **0.94** | **0.55** | **0.97** | **0.92** | **0.41** | **0.78** | **0.56** | **0.55** | **0.62** | **0.53** |
|  |  | 1 | **0.90** | **0.52** | **0.97** | **0.91** | **0.43** | **0.79** | **0.57** | **0.52** | **0.61** | **0.53** |
|  |  | 2 | **0.81** | **0.48** | **0.95** | **0.90** | **0.43** | **0.79** | **0.56** | **0.57** | **0.61** | **0.58** |
|  |  | 3 | **0.81** | **0.42** | **0.95** | **0.88** | **0.43** | **0.79** | **0.53** | **0.60** | **0.58** | **0.58** |
|  | Fixed | None | **0.97** | **0.53** | **0.98** | **0.93** | **0.43** | **0.61** | **0.55** | **0.59** | **0.64** | **0.62** |
|  |  | 1 | **0.96** | **0.51** | **0.97** | **0.91** | **0.45** | **0.60** | **0.56** | **0.52** | **0.59** | **0.54** |
|  |  | 2 | **0.92** | **0.47** | **0.96** | **0.89** | **0.46** | **0.60** | **0.55** | **0.57** | **0.59** | **0.57** |
|  |  | 3 | **0.85** | **0.48** | **0.95** | **0.91** | **0.46** | **0.59** | **0.53** | **0.57** | **0.58** | **0.54** |

Table S5: Concurvity estimates for the **Hillsborough Bay** Generalized Additive Models. Values range from 0 to 1 and assess the degree of multicollinearity among predictors. Results are shown for models with different total nitrogen loading lags, whether salinity or loading were corrected for seasonality, and constraints on the number of smoothers (K) for time, loading, and salinity (flexible or fixed). Loads were evaluated as cumulative sums for the current month up to a three month lag. Font size and shading are proportional among cells to emphasize differences.

| Seasonal Correction | K | TN Load Lag | s(dec_time) | s(doy) | s(sal) | s(tn_load) | ti(dec_time,doy) | ti(dec_time,sal) | ti(sal,doy) | ti(dec_time,tn_load) | ti(tn_load,doy) | ti(tn_load,sal) |
| --- | --- | --- | --- | --- | --- | --- | --- | --- | --- | --- | --- | --- |
| No | Flexible | None | **0.88** | **0.63** | **0.98** | **0.99** | **0.45** | **0.67** | **0.71** | **0.71** | **0.84** | **0.85** |
|  |  | 1 | **0.89** | **0.63** | **0.99** | **0.99** | **0.48** | **0.79** | **0.77** | **0.84** | **0.87** | **0.93** |
|  |  | 2 | **0.96** | **0.62** | **0.99** | **0.99** | **0.51** | **0.78** | **0.79** | **0.82** | **0.85** | **0.93** |
|  |  | 3 | **0.93** | **0.64** | **0.99** | **0.99** | **0.56** | **0.75** | **0.79** | **0.80** | **0.84** | **0.89** |
|  | Fixed | None | **0.98** | **0.59** | **0.98** | **0.99** | **0.45** | **0.65** | **0.70** | **0.74** | **0.85** | **0.88** |
|  |  | 1 | **0.97** | **0.58** | **0.99** | **0.98** | **0.48** | **0.76** | **0.78** | **0.86** | **0.88** | **0.93** |
|  |  | 2 | **0.97** | **0.59** | **0.99** | **0.99** | **0.53** | **0.76** | **0.79** | **0.84** | **0.86** | **0.93** |
|  |  | 3 | **0.97** | **0.60** | **0.98** | **0.99** | **0.55** | **0.74** | **0.79** | **0.80** | **0.85** | **0.91** |
| Yes | Flexible | None | **0.97** | **0.52** | **0.95** | **0.94** | **0.41** | **0.73** | **0.48** | **0.50** | **0.62** | **0.55** |
|  |  | 1 | **0.91** | **0.50** | **0.95** | **0.97** | **0.44** | **0.75** | **0.52** | **0.66** | **0.74** | **0.69** |
|  |  | 2 | **0.94** | **0.50** | **0.96** | **0.96** | **0.47** | **0.75** | **0.56** | **0.68** | **0.71** | **0.70** |
|  |  | 3 | **0.96** | **0.50** | **0.97** | **0.96** | **0.50** | **0.77** | **0.60** | **0.70** | **0.72** | **0.72** |
|  | Fixed | None | **0.98** | **0.52** | **0.98** | **0.96** | **0.43** | **0.58** | **0.48** | **0.57** | **0.62** | **0.56** |
|  |  | 1 | **0.95** | **0.50** | **0.97** | **0.96** | **0.47** | **0.60** | **0.52** | **0.68** | **0.73** | **0.71** |
|  |  | 2 | **0.96** | **0.51** | **0.97** | **0.94** | **0.49** | **0.60** | **0.56** | **0.71** | **0.74** | **0.70** |
|  |  | 3 | **0.98** | **0.50** | **0.98** | **0.92** | **0.51** | **0.61** | **0.61** | **0.70** | **0.74** | **0.72** |

Table S6: Concurvity estimates for the **Middle Tampa Bay** Generalized Additive Models. Values range from 0 to 1 and assess the degree of multicollinearity among predictors. Results are shown for models with different total nitrogen loading lags, whether salinity or loading were corrected for seasonality, and constraints on the number of smoothers (K) for time, loading, and salinity (flexible or fixed). Loads were evaluated as cumulative sums for the current month up to a three month lag. Font size and shading are proportional among cells to emphasize differences.

| Seasonal Correction | K | TN Load Lag | s(dec_time) | s(doy) | s(sal) | s(tn_load) | ti(dec_time,doy) | ti(dec_time,sal) | ti(sal,doy) | ti(dec_time,tn_load) | ti(tn_load,doy) | ti(tn_load,sal) |
| --- | --- | --- | --- | --- | --- | --- | --- | --- | --- | --- | --- | --- |
| No | Flexible | None | **0.98** | **0.68** | **0.98** | **0.97** | **0.54** | **0.67** | **0.60** | **0.74** | **0.82** | **0.75** |
|  |  | 1 | **0.91** | **0.67** | **0.98** | **0.98** | **0.59** | **0.72** | **0.68** | **0.74** | **0.85** | **0.80** |
|  |  | 2 | **0.87** | **0.67** | **0.97** | **0.98** | **0.59** | **0.74** | **0.71** | **0.70** | **0.83** | **0.76** |
|  |  | 3 | **0.86** | **0.65** | **0.97** | **0.98** | **0.59** | **0.76** | **0.72** | **0.69** | **0.79** | **0.78** |
|  | Fixed | None | **0.99** | **0.61** | **0.98** | **0.97** | **0.55** | **0.58** | **0.59** | **0.76** | **0.83** | **0.78** |
|  |  | 1 | **0.97** | **0.61** | **0.98** | **0.97** | **0.58** | **0.64** | **0.66** | **0.76** | **0.86** | **0.81** |
|  |  | 2 | **0.93** | **0.61** | **0.98** | **0.98** | **0.59** | **0.69** | **0.70** | **0.69** | **0.83** | **0.76** |
|  |  | 3 | **0.90** | **0.61** | **0.98** | **0.99** | **0.59** | **0.71** | **0.71** | **0.68** | **0.80** | **0.76** |
| Yes | Flexible | None | **0.98** | **0.61** | **0.97** | **0.94** | **0.39** | **0.76** | **0.49** | **0.65** | **0.66** | **0.55** |
|  |  | 1 | **0.93** | **0.56** | **0.93** | **0.96** | **0.40** | **0.77** | **0.51** | **0.63** | **0.66** | **0.57** |
|  |  | 2 | **0.90** | **0.52** | **0.94** | **0.96** | **0.42** | **0.78** | **0.53** | **0.62** | **0.66** | **0.64** |
|  |  | 3 | **0.84** | **0.46** | **0.92** | **0.96** | **0.43** | **0.79** | **0.55** | **0.69** | **0.67** | **0.67** |
|  | Fixed | None | **0.99** | **0.56** | **0.98** | **0.94** | **0.43** | **0.58** | **0.50** | **0.73** | **0.73** | **0.66** |
|  |  | 1 | **0.96** | **0.53** | **0.94** | **0.93** | **0.45** | **0.61** | **0.52** | **0.66** | **0.66** | **0.58** |
|  |  | 2 | **0.96** | **0.51** | **0.95** | **0.92** | **0.45** | **0.61** | **0.52** | **0.61** | **0.66** | **0.59** |
|  |  | 3 | **0.88** | **0.47** | **0.92** | **0.95** | **0.46** | **0.62** | **0.55** | **0.67** | **0.67** | **0.62** |

Table S7: Concurvity estimates for the **Lower Tampa Bay** Generalized Additive Models. Values range from 0 to 1 and assess the degree of multicollinearity among predictors. Results are shown for models with different total nitrogen loading lags, whether salinity or loading were corrected for seasonality, and constraints on the number of smoothers (K) for time, loading, and salinity (flexible or fixed). Loads were evaluated as cumulative sums for the current month up to a three month lag. Font size and shading are proportional among cells to emphasize differences.

| Seasonal Correction | K | TN Load Lag | s(dec_time) | s(doy) | s(sal) | s(tn_load) | ti(dec_time,doy) | ti(dec_time,sal) | ti(sal,doy) | ti(dec_time,tn_load) | ti(tn_load,doy) | ti(tn_load,sal) |
| --- | --- | --- | --- | --- | --- | --- | --- | --- | --- | --- | --- | --- |
| No | Flexible | None | **0.92** | **0.66** | **0.95** | **0.96** | **0.53** | **0.72** | **0.64** | **0.61** | **0.76** | **0.69** |
|  |  | 1 | **0.84** | **0.67** | **0.91** | **0.97** | **0.61** | **0.74** | **0.69** | **0.65** | **0.79** | **0.67** |
|  |  | 2 | **0.80** | **0.65** | **0.91** | **0.97** | **0.63** | **0.74** | **0.71** | **0.62** | **0.76** | **0.66** |
|  |  | 3 | **0.77** | **0.65** | **0.91** | **0.96** | **0.63** | **0.77** | **0.72** | **0.63** | **0.76** | **0.68** |
|  | Fixed | None | **0.98** | **0.61** | **0.96** | **0.95** | **0.53** | **0.65** | **0.65** | **0.64** | **0.79** | **0.70** |
|  |  | 1 | **0.91** | **0.62** | **0.92** | **0.98** | **0.61** | **0.68** | **0.69** | **0.65** | **0.80** | **0.69** |
|  |  | 2 | **0.85** | **0.62** | **0.90** | **0.97** | **0.62** | **0.70** | **0.71** | **0.61** | **0.76** | **0.66** |
|  |  | 3 | **0.82** | **0.61** | **0.90** | **0.97** | **0.62** | **0.71** | **0.71** | **0.64** | **0.77** | **0.70** |
| Yes | Flexible | None | **0.93** | **0.57** | **0.94** | **0.95** | **0.37** | **0.72** | **0.46** | **0.57** | **0.65** | **0.57** |
|  |  | 1 | **0.90** | **0.52** | **0.90** | **0.96** | **0.39** | **0.73** | **0.48** | **0.62** | **0.66** | **0.61** |
|  |  | 2 | **0.90** | **0.49** | **0.89** | **0.97** | **0.39** | **0.73** | **0.45** | **0.63** | **0.72** | **0.63** |
|  |  | 3 | **0.93** | **0.47** | **0.89** | **0.96** | **0.42** | **0.74** | **0.46** | **0.67** | **0.69** | **0.65** |
|  | Fixed | None | **0.98** | **0.55** | **0.94** | **0.96** | **0.41** | **0.61** | **0.47** | **0.67** | **0.71** | **0.63** |
|  |  | 1 | **0.97** | **0.52** | **0.95** | **0.96** | **0.43** | **0.63** | **0.49** | **0.63** | **0.69** | **0.66** |
|  |  | 2 | **0.95** | **0.49** | **0.87** | **0.97** | **0.42** | **0.66** | **0.46** | **0.64** | **0.72** | **0.63** |
|  |  | 3 | **0.95** | **0.49** | **0.87** | **0.97** | **0.43** | **0.67** | **0.47** | **0.65** | **0.68** | **0.65** |

Table S8: Contemporary loading trends from 2012 to 2024 by bay segment. Results are for regression models of annual loading with year. OTB: Old Tampa Bay; HB: Hillsborough Bay; MTB: Middle Tampa Bay; LTB: Lower Tampa Bay.

| Bay Segment | R.sq. | Year Estimate | Std. Error | p |
| --- | --- | --- | --- | --- |
| OTB | 0.05 | -9.39 | 12.80 | 0.479 |
| HB | 0.01 | 6.13 | 22.94 | 0.794 |
| MTB | 0.00 | 2.76 | 13.40 | 0.840 |
| LTB | 0.14 | 5.29 | 3.92 | 0.204 |

## References

Akaike, H. 1974. A new look at the statistical model identification. *IEEE Transactions on Automatic Control* 19. IEEE: 716–723. <https://doi.org/10.1109/TAC.1974.1100705>.
